# Evolutionary Patterns among Living and Fossil Kogiid Sperm Whales: Evidence from the Neogene of Central America

**DOI:** 10.1371/journal.pone.0123909

**Published:** 2015-04-29

**Authors:** Jorge Velez-Juarbe, Aaron R. Wood, Carlos De Gracia, Austin J. W. Hendy

**Affiliations:** 1 Department of Mammalogy, Natural History Museum of Los Angeles County, Los Angeles, California, United States of America; 2 John D. Cooper Archaeological and Paleontological Center, Department of Geological Sciences, California State University, Fullerton, California, United States of America; 3 Department of Geological and Atmospheric Sciences, Iowa State University, Ames, Iowa, United States of America; 4 Smithsonian Tropical Research Institute, Balboa-Ancon, Panama; 5 Department of Invertebrate Paleontology, Natural History Museum of Los Angeles County, Los Angeles, California, United States of America; Team 'Evo-Devo of Vertebrate Dentition', FRANCE

## Abstract

Kogiids are known by two living species, the pygmy and dwarf sperm whale (*Kogia breviceps* and *K*. *sima*). Both are relatively rare, and as their names suggest, they are closely related to the sperm whale, all being characterized by the presence of a spermaceti organ. However, this organ is much reduced in kogiids and may have become functionally different. Here we describe a fossil kogiid from the late Miocene of Panama and we explore the evolutionary history of the group with special attention to this evolutionary reduction. The fossil consists of cranial material from the late Tortonian (~7.5 Ma) Piña facies of the Chagres Formation in Panama. Detailed comparison with other fossil and extant kogiids and the results of a phylogenetic analysis place the Panamanian kogiid, herein named *Nanokogia isthmia* gen. et sp. nov., as a taxon most closely related to *Praekogia cedrosensis* from the Messinian (~6 Ma) of Baja California and to *Kogia* spp. Furthermore our results show that reduction of the spermaceti organ has occurred iteratively in kogiids, once in *Thalassocetus antwerpiensis* in the early-middle Miocene, and more recently in *Kogia* spp. Additionally, we estimate the divergence between extant species of *Kogia* at around the late Pliocene, later than previously predicted by molecular estimates. Finally, comparison of *Nanokogia* with the coeval *Scaphokogia cochlearis* from Peru shows that these two species display a greater morphological disparity between them than that observed between the extant members of the group. We hypothesize that this reflects differences in feeding ecologies of the two species, with *Nanokogia* being more similar to extant *Kogia*. *Nanokogia* shows that kogiids have been part of the Neotropical marine mammal communities at least since the late Miocene, and gives us insight into the evolutionary history and origins of one of the rarest groups of living whales.

## Introduction

Kogiidae is a family of toothed whales represented by two extant species, the pygmy (*Kogia breviceps* (Blainville [1])) and the dwarf (*K*. *sima* (Owen [2])) sperm whales, and having a worldwide distribution from temperate to tropical regions [3–4]. However, they are some of the rarest whales, and hence little is known of their life history [5]. This is also true of their fossil record, which is relatively sparse, with a total of five species described so far: *Thalassocetus antwerpiensis* Abel [6], from the early-middle Miocene of Belgium [7]; *Scaphokogia cochlearis* Muizon [8], from the late Miocene (Tortonian) of Peru [9–10]; *Praekogia cedrosensis* Barnes [11], from the late Miocene (Messinian) of Baja California; *Aprixokogia kelloggi* Whitmore and Kaltenbach [12], from the early Pliocene (Zanclean) of North Carolina; and *Kogia pusilla* (Pilleri [13]), from the late Pliocene (Piacenzian) of Italy [14]. In addition to these, there are a number of additional Neogene records of kogiids based on family-level diagnostic elements such as isolated earbones (e.g. [15]), showing that the group was already widespread throughout the Neogene in subtropical to temperate regions. Nonetheless, we still know very little about their ancient diversity and distribution due to the scarcity of cranial material described, as well as the limited taxonomic information provided by other elements.

**Fig 1:**
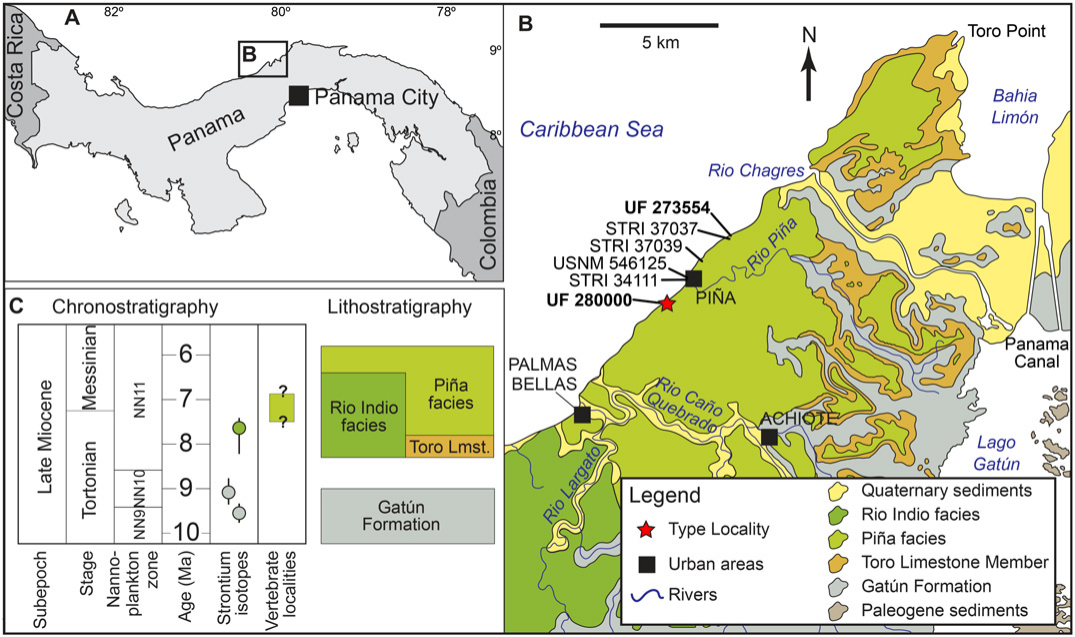
Locality map. A, map of Central America showing the localities. B, map of central Panama, showing the distribution of the Chagres Formation as well as the localities of fossil cetaceans mentioned in the text. C, chronostratigraphic and lithostratigraphic relationships of the Chagres Formation (modified from Hendy et al. [17]).

Here we describe the first fossil kogiid from the Central American and Caribbean region, based on cranial material recovered from the late Miocene (latest Tortonian) Chagres Formation [16–17] on the Caribbean coast of Panama (Fig 1). The Chagres kogiid represents a new taxon closely related to *Praekogia cedrosensis* and *Kogia* spp. The other known Neotropic fossil kogiid is *Scaphokogia cochlearis* from similar-aged (Tortonian) deposits in Peru. These two occurrences show that kogiids have been established in the Neotropics at least since that time. However, the new Panamanian taxon and *Scaphokogia cochlearis* display much greater morphological disparity between them, relative to what is seen in extant *Kogia* spp., clearly showing that we are far from fully understanding deep-time diversity in kogiids. The new taxon from Panama is added to an increasing list of fossil marine mammals from the region, and it is a step further towards a better understanding of deep-time diversity of Neotropical marine mammals.

## Materials and Methods

Measurements of the skulls and mandible (Tables 1 and 2) follow those outlined by Perrin [18], while the morphological terminology follows Mead and Fordyce [19]. The chronostratigraphy used here follows that of Cohen et al. [20]. For the phylogenetic analysis we followed Lambert et al. [21] and throughout the text we have cross-referenced the morphology of specimens with the corresponding character states for ease of comparison and clarity, e.g. (c. 6[0]) refers to state 0 of character 6.

**Table 1:** Measurements (in mm) of skulls of *Nanokogia isthmia* gen. et sp. nov. (modified from Perrin [18]).

	UF 280000	UF 273554
Condylobasal length	348	-
Length of rostrum	178	-
Width of rostrum at base	129	-
Width of rostrum at 60 mm anterior to line across hindmost limits of antorbital notches	117	-
Width of rostrum at midlength	85	-
Width of premaxillae at midlength of rostrum	46	-
Width of rostrum at ¾ length, measured from posterior end	49	-
Distance from tip of rostrum to external nares	181	-
Greatest preorbital width (width across preorbital processes)	-	228e
Greatest postorbital width	190+	228e
Least supraorbital width	184	214e
Maximum width of external nares	48	30
Greatest width across zygomatic processes of squamosal	220	223
Greatest width of premaxillae	90	-
Greatest parietal width, within posttemporal fossae	150	-
Vertical external height of braincase from midline of basisphenoid to summit of supraoccipital, but not including supraoccipital crest	110	112
Greatest length of posttemporal fossa, measured to external margin of raised suture	79	84
Greatest width of posttemporal fossa at right angles to greatest length	43	51
Length of left orbit—from apex of preorbital process of frontal to apex of postorbital process	-	72
Length of antorbital process of left lacrimal	58	-
Greatest width of internal nares	35	35
Greatest length of pterygoid	122	96+
Maximum width across occipital condyles	72	73
Height of foramen magnum	33	38
Width of foramen magnum	33	33

Abbreviations: e = estimate; + = measurement on incomplete element.

**Table 2:** Measurements (in mm) of mandible (UF 280000) of *Nanokogia isthmia* gen. et sp. nov. (modified from Perrin [18]).

Length of left toothrow	178
Greater length of left ramus	222+
Greatest height of left ramus	51+
Length of left mandibular fossa	41+
Length from anterior tip to mandibular canal	180
Length of symphysis	116
Maximum height of symphysis	18
Number of teeth—left	14
Number of teeth—right	10+

**Abbreviations**: **+** = measurement on incomplete element.

### Fieldwork

Permits for fieldwork in Piña and other parts of Panama were obtained from the Dirección de Recursos Minerales de Panamá. Fieldwork only involved geological and paleontological sampling and collecting; it did not involve endangered or protected species. The specific coordinates of the field sites are provided in the Results section. Export of specimens was possible through a permit issued by the Ministerio de Comercio e Industria de la República de Panamá to the Smithsonian Tropical Research Institute.

### Specimens Observed

We compared the Panamanian material with specimens from the mammalogy collections at the Natural History Museum of Los Angeles County (LACM), Florida Museum of Natural History (UF) and National Museum of Natural History (USNM) for the following taxa: *Kogia breviceps* (LACM 27082, LACM 95745, UF 13562, UF 14213, UF 14214, UF 17532, UF 18702, UF 18704, UF 19128, UF 25545; USNM 504902, USNM 504921), and *Kogia sima* (LACM 47142, LACM 95817, UF 18705, UF 18706, UF 24629, UF 25573, UF 25575–25578). We also made comparisons with fossil taxa from the following collections: Muséum National d’Histoire Naturelle (MNHN) for *Scaphokogia cochlearis* (MNHN PPI 229); Museo de Historia Natural de la Universidad Nacional Mayor de San Marcos (MUSM) for *Livyatan melvillei* (MUSM 1676); Natural History Museum of Los Angeles County and National Museum of Natural History for *Aprixokogia kelloggi* (LACM 117744 [cast] and USNM 187015), and Museum of Paleontology, University of California (UCMP) for *Praekogia cedrosensis* (UCMP 315229).

### Nomenclatural Acts

The electronic edition of this article conforms to the requirements of the amended International Code of Zoological Nomenclature, and hence the new names contained herein are available under that Code from the electronic edition of this article. This published work and the nomenclatural acts it contains have been registered in ZooBank, the online registration system for the ICZN. The ZooBank LSIDs (Life Science Identifiers) can be resolved and the associated information viewed through any standard web browser by appending the LSID to the prefix "http://zoobank.org/". The LSID for this publication is: urn:lsid:zoobank.org:pub:ADA83315-CCAC-4E6C-B7D2-90BC77D2F044. The electronic edition of this work was published in a journal with an ISSN, and has been archived and is available from the following digital repositories: PubMed Central, LOCKSS.

## Results

### Systematic Paleontology

Mammalia Linnaeus, 1758 [22]

Cetacea Brisson, 1762 [23]

Odontoceti Flower, 1867 [24]

Pan-Physeteroidea new clade name

Physeteroidea Gray, 1821 [25]

Kogiidae Gill, 1871 [26]

*Nanokogia* gen. nov. urn:lsid:zoobank.org:act:D2BEAD8F-D882-44F3-85EE-6BE6E601AEF7

#### Type and only known species

*Nanokogia isthmia* gen. et sp. nov.

#### Etymology

The name ‘*Nanokogia*’ is formed by the combination of ‘*nano*’, from the Latin “*nanus*” which means dwarf, in reference to the small size of the skull and estimated total length of the body, relative to most other kogiids, with ‘*Kogia*’, fem., which is the genus name of the extant members of the group and a widely used suffix for other fossil taxa of the group.

#### Range

Late Miocene (latest Tortonian-early Messinian [17]) of Panama.

#### Diagnosis

Same as that for the type species until other species are described.

*Nanokogia isthmia* sp. nov. urn:lsid:zoobank.org:act:25FB8526-74BA-4A03-A80A-8DECD9DB2234

#### Holotype

UF 280000, nearly complete adult skull and mandible; missing teeth, ear bones, and a portion of the left side of the cranium, and part of the right horizontal ramus. Collected by J. Velez-Juarbe, December 12, 2013.

#### Etymology

The specific name ‘*isthmia*’, derives from the Latin ‘*isthmus*’ in reference to the Isthmus of Panama.

#### Type locality

Piña 1 (UF locality YPA089), about 1.2 km southwest of the village of Piña, Colón Province, Panama (9.27326°N, 80.05454°W) (Fig 1A and 1C).

#### Formation and age

Chagres Formation; late Miocene (latest Tortonian-early Messinian).

#### Referred specimen

UF 273554, adult skull, missing rostrum and part of the right side of the cranium; Chagres Formation, Piña 2 (UF locality YPA087), about 100 m northeast of mouth of Quebrada La Toba, Piña, Colón Province, Panama (09.29338°N, 80.03772°W) (Fig 1B); collected by C. De Gracia, July 5, 2012.

#### Range

Known from the upper Miocene of Panama.

#### Differential diagnosis

Small kogiids, with an estimated body length of ~1.95–2.16 m (based on equation for Physeteroidea from Pyenson and Sponberg [27]), similar in size to *Kogia sima* [28]. Recognized as kogiid based on: bizygomatic width of less than 40 cm (c. 8[0]); presence of a sagittal crest; c. 14[1]); external nares greatly asymmetric; c. 18[1]) and located at the level of the supraorbital processes; absence of nasals (c. 19[2]); and right maxilla reaching the sagittal plane of the skull on the posterior wall of the supracranial basin. Differs from all other kogiids by the following combination of characters: absence of upper teeth, shared with *Scaphokogia cochlearis* and *Kogia* spp., unknown in *Praekogia cedrosensis* and *Thalassocetus antwerpiensis*; antorbital notches form a narrow slit (c. 9[2]), shared with *Scaphokogia*, *Praekogia*, and *Kogia*; postorbital process overhanging the zygomatic process, shared with *Thalassocetus antwerpiensis* and *Kogia*; presphenoid not covered ventrally by the vomer, shared with *Aprixokogia kelloggi*, *Scaphokogia*, and *Kogia*; left premaxilla not reaching the sagittal facial crest, shared with *Aprixokogia*, *Scaphokogia* and *Kogia*. Shares with *Praekogia* and *Kogia*: antorbital notches within the supracranial basin (c. 10[1]); long postglenoid process of the squamosal (c. 28[0]); and, a wide notch on the squamosal for the enlarged posterior process of the tympanic (c. 29[1]). Shares with *Aprixokogia* an elongated temporal fossa (c. 26[0]) in contrast to the anteroposteriorly-shortened fossa of *Kogia*, or the rounded fossa in *Praekogia*. Shares with *Praekogia* relatively mediolaterally-thin maxillary crests; and wide but relatively shallow supracranial basin, shared with *Praekogia* and *Thalassocetus*, unlike the deeper basin seen in *Aprixokogia* and *Kogia*, or the broad, rounded basin of *Scaphokogia*. Shares with *Kogia* the presence of a narrow, rounded notch between the hamular process and the medial lamina of the pterygoid (absent in *Aprixokogia*, unknown in *Thalassocetus*, *Scaphokogia* and *Praekogia*). Differs further from *Kogia sima* and *K*. *breviceps* in having a relatively longer rostrum, similar to *K*. *pusilla*. Diagnosed by the following autapomorphies: in lateral view the posterodorsal corner of the lacrimal + jugal is not deeply wedged between the frontal and maxilla (c. 23[0]); the lateral edge of the frontal portion of the right premaxilla is convex, not forming a crest; and the nuchal crest and posterolateral edges of the supracranial basin overhang the occipital surface of the cranium in dorsal, lateral and posterior views, but not to the extreme seen in *Scaphokogia*.

## Description

### Skull

**Fig 2:**
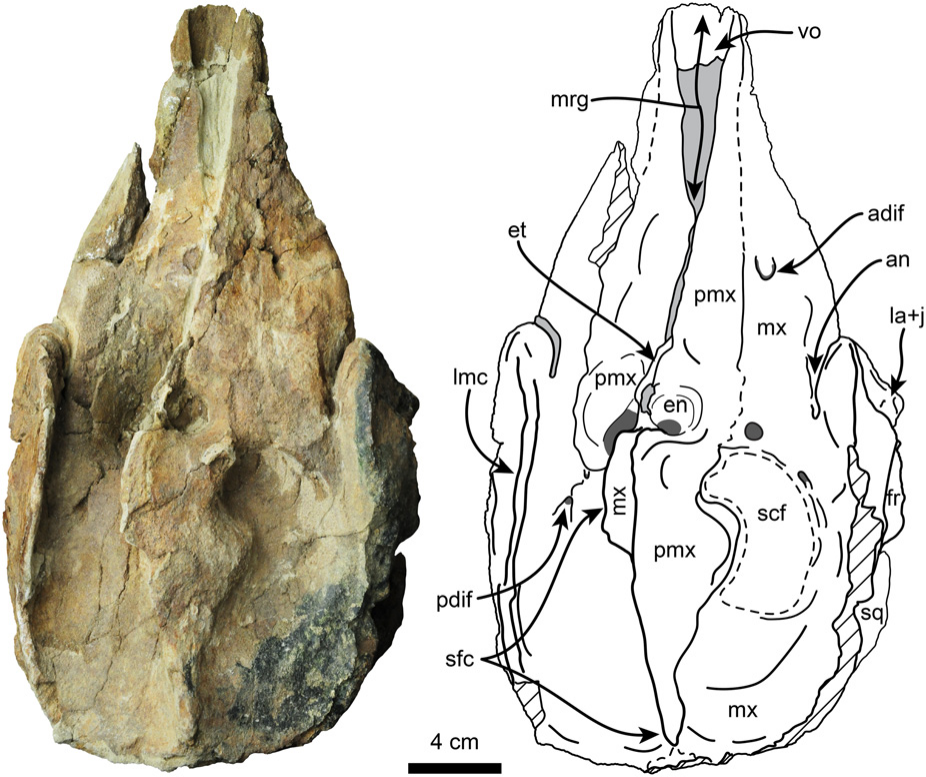
Dorsal view of holotype skull of *Nanokogia isthmia* gen. et sp. nov. (UF 280000). Abbreviations: adif, anterior dorsal infraorbital foramen; an, antorbital notch; en, external nares; et, ethmoid; fr, frontal; la+j, lacrimal + jugal; lmc, lateral maxillary crest; mrg, mesorostral groove; mx, maxilla; pmx, premaxilla; pdif, posterior dorsal infraorbital foramen; scf, supracranial fossa; sfc, sagittal facial crest; sq, squamosal; vo, vomer. Gray shaded areas indicate sediment; diagonal lines denote broken surfaces. Gray shaded areas indicate sediment; diagonal lines denote broken surfaces.

**Fig 3:**
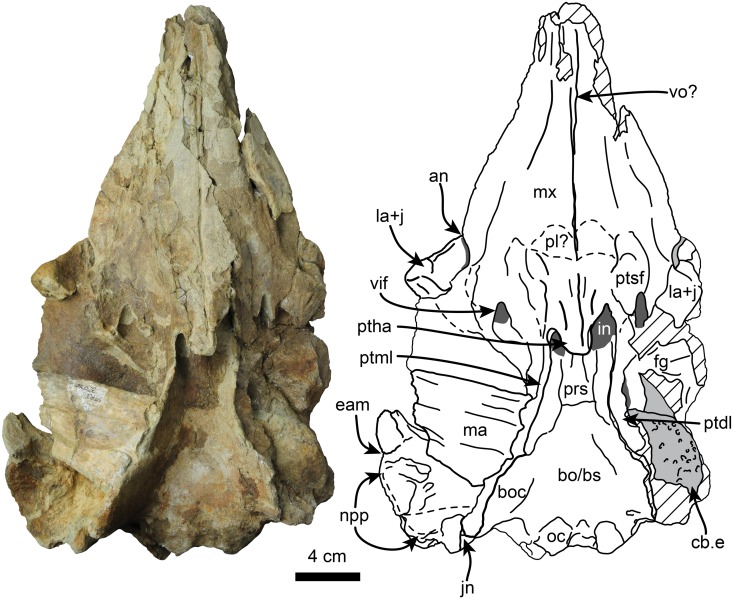
Ventral view of holotype skull of *Nanokogia isthmia* gen. et sp. nov. (UF 280000). Abbreviations: an, antorbital notch; bo/bs, basioccipital/basisphenoid; boc, basioccipital crest; cb.e, cerebral endocast; eam, external auditory meatus; fg, frontal groove; in, internal nares; jn, jugular notch; la+j, lacrimal + jugal; ma, mandible; mx, maxilla; npp, notch for posterior process of tympanic; oc, occipital condyles; pl, palatine; prs, presphenoid; ptdl, dorsal lamina of pterygoid; ptha, pterygoid hamulus; ptml, medial lamina of pterygoid; ptsf, pterygoid sinus fossa; tsr, tympanosquamosal recess; vif, ventral infraorbital foramen; vo, vomer. Gray shaded areas indicate sediment; diagonal lines denote broken surfaces.

**Fig 4:**
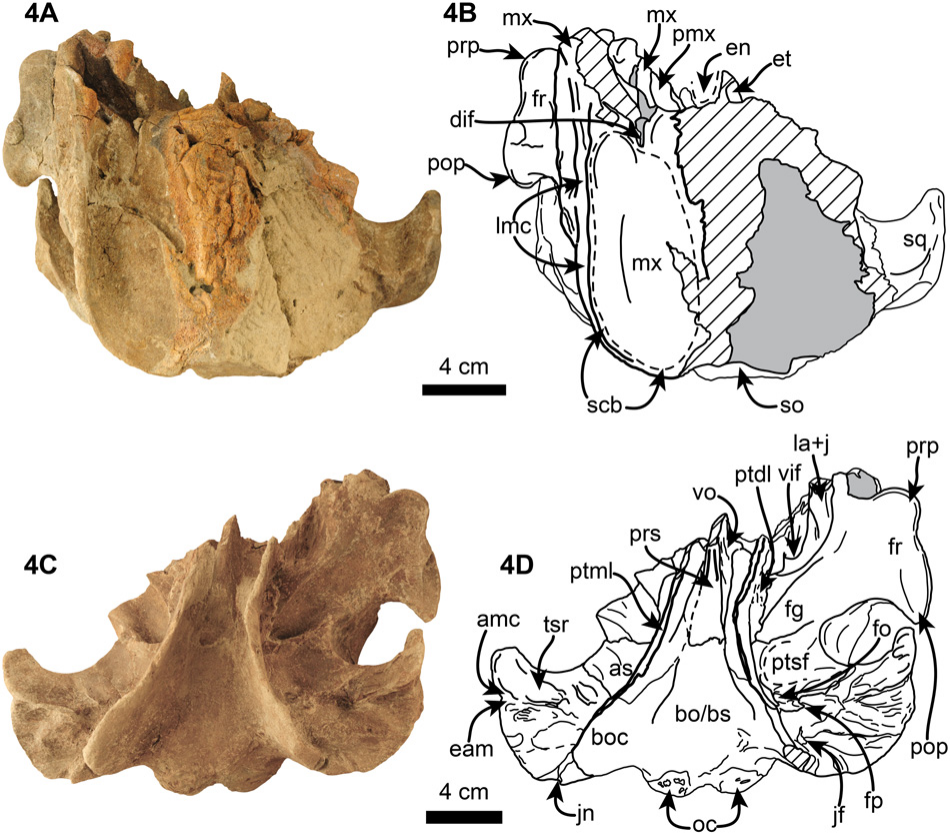
Dorsal, 4A-B, and ventral, 4C-D, views of referred specimen of *Nanokogia isthmia* gen. et sp. nov. (UF 273554). Abbreviations: amc, anterior meatal crest; as, alisphenoid; bo, basioccipital; boc, basioccipital crest; dif, dorsal infraorbital foramen; eam, external auditory meatus; en, external nares; fg, frontal groove; fo, foramen ovale; fp, falciform process of squamosal; fr, frontal; jf, jugular foramen; jn, jugular notch; la+j, lacrimal + jugal; lmc, lateral maxillary crest; mx, maxilla; oc, occipital condyles; pmx, premaxilla; pop, postorbital process; prp, preorbital process; prs, presphenoid; ptdl, dorsal lamina of pterygoid; ptml, medial lamina of pterygoid; ptsf, pterygoid sinus fossa; scb, supracranial basin; so, supraoccipital; sq, squamosal; tsr, tympanosquamosal recess; vif, ventral infraorbital foramen; vo, vomer. Gray shaded areas indicate sediment; diagonal lines denote broken surfaces.

**Fig 5:**
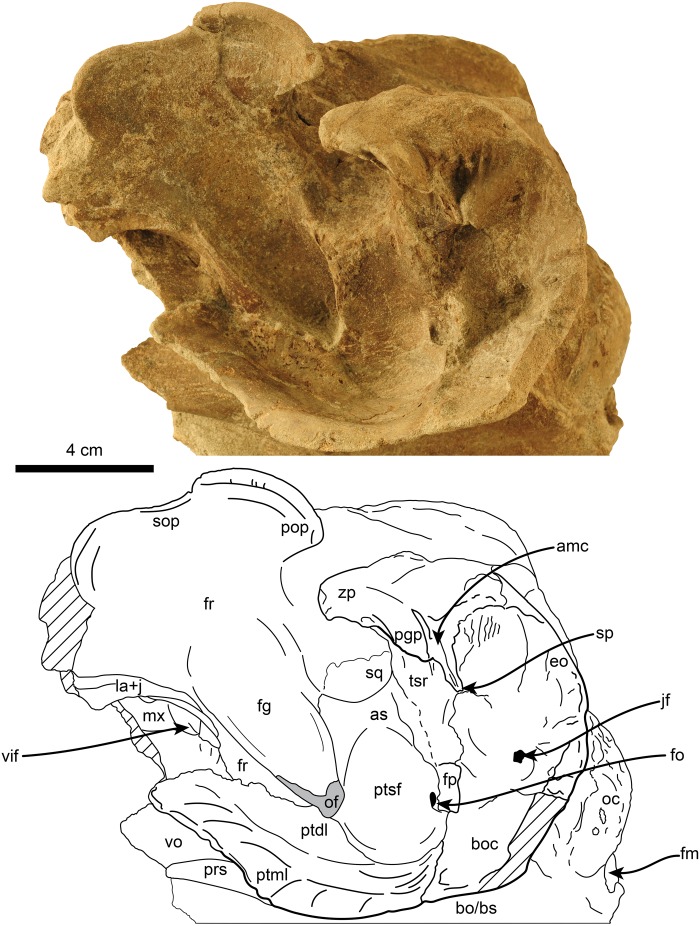
Left ventrolateral view of referred specimen of *Nanokogia isthmia* gen. et sp. nov. (UF 273554). Abbreviations: amc, anterior meatal crest; as, alisphenoid; bo/bs, basioccipital/basisphenoid; boc, basioccipital crest; eo, exoccipital; fg, frontal groove; fm, foramen magnum; fo, foramen ovale; fp, falciform process of squamosal; fr, frontal; jf, jugular foramen; la+j, lacrimal + jugal; mx, maxilla; oc, occipital condyle; of, optic foramen; pgp, postglenoid process; pop, postorbital process; prs, presphenoid; ptdl, dorsal lamina of pterygoid; ptml, medial lamina of pterygoid; ptsf, pterygoid sinus fossa; sop, supraorbital process; sp, spiny process; sq, squamosal; tsr, tympanosquamosal recess; vif, ventral infraorbital foramen; vo, vomer; zp, zygomatic process. Gray shaded areas indicate sediment; diagonal lines denote broken surfaces.

**Fig 6:**
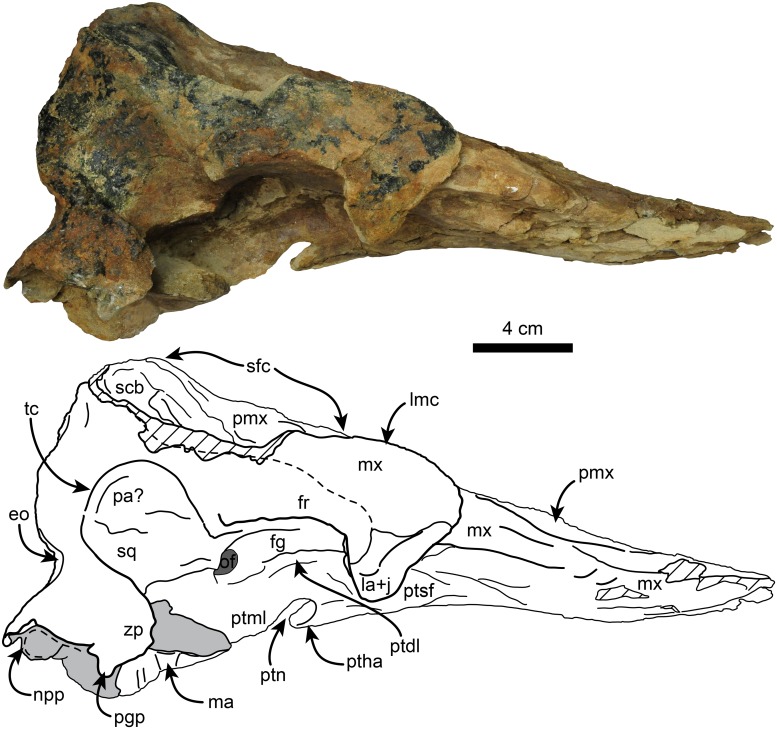
Right lateral view of holotype skull of *Nanokogia isthmia* gen. et sp. nov. (UF 280000). Abbreviations: eo, exoccipital; fg, frontal groove; fr, frontal; la+j, lacrimal + jugal; ma, mandible; mx, maxilla; lmc, lateral maxillary crest; npp, notch for posterior process of tympanic; of, optic foramen; pa, parietal; pgp, postglenoid process; pmx, premaxilla; ptdl, dorsal lamina of pterygoid; ptha, pterygoid hamulus; ptml, medial lamina of pterygoid; ptn, pterygoid notch; ptsf, pterygoid sinus fossa; scb, supracranial basin; sfc, sagittal facial crest; sq, squamosal; tc, temporal crest; zp, zygomatic process. Gray shaded areas indicate sediment; diagonal lines denote broken surfaces.

**Fig 7:**
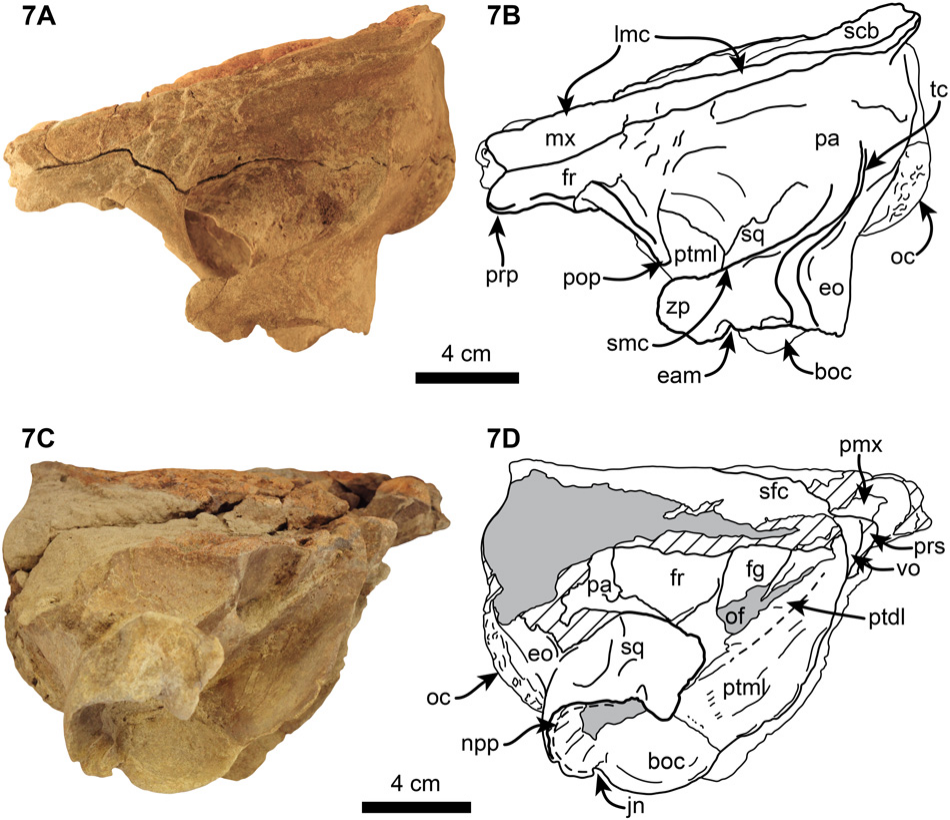
Left, 6A-B, and right, 6C-D, lateral views of referred specimen of *Nanokogia isthmia* gen. et sp. nov. (UF 273554). Abbreviations: boc, basioccipital crest; eam, external auditory meatus; eo, exoccipital; fr, frontal; lmc, lateral maxillary crest; mx, maxilla; npp, notch for posterior process of tympanic; oc, occipital condyle; of, optic foramen; pa, parietal; pmx, premaxilla; pop, postorbital process; prp, preorbital process; prs, presphenoid; ptdl, dorsal lamina of pterygoid; ptml, medial lamina of pterygoid; scb, supracranial basin; smc, supramastoid crest; sq, squamosal; tc, temporal crest; vo, vomer; zp, zygomatic process. Gray shaded areas indicate sediment; diagonal lines denote broken surfaces.

**Fig 8:**
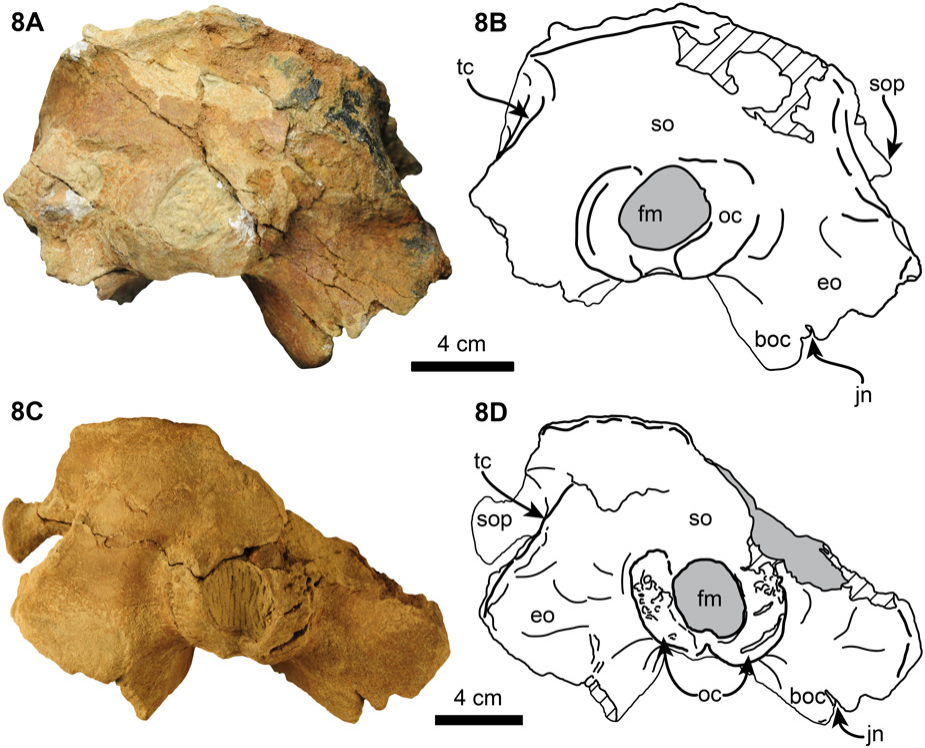
Posterior views of holotype (UF 280000), 7A-B, and referred specimen (UF 273554), 7CD, skulls of *Nanokogia isthmia* gen. et sp. nov. Abbreviations: boc, basioccipital crest; eo, exoccipital; fm, foramen magnum; jn, jugular notch; oc, occipital condyles; so, supraoccipital; sop, supraorbital process of frontal; tc, temporal crest. Gray shaded areas indicate sediment; diagonal lines denote broken surfaces.

**Fig 9:**
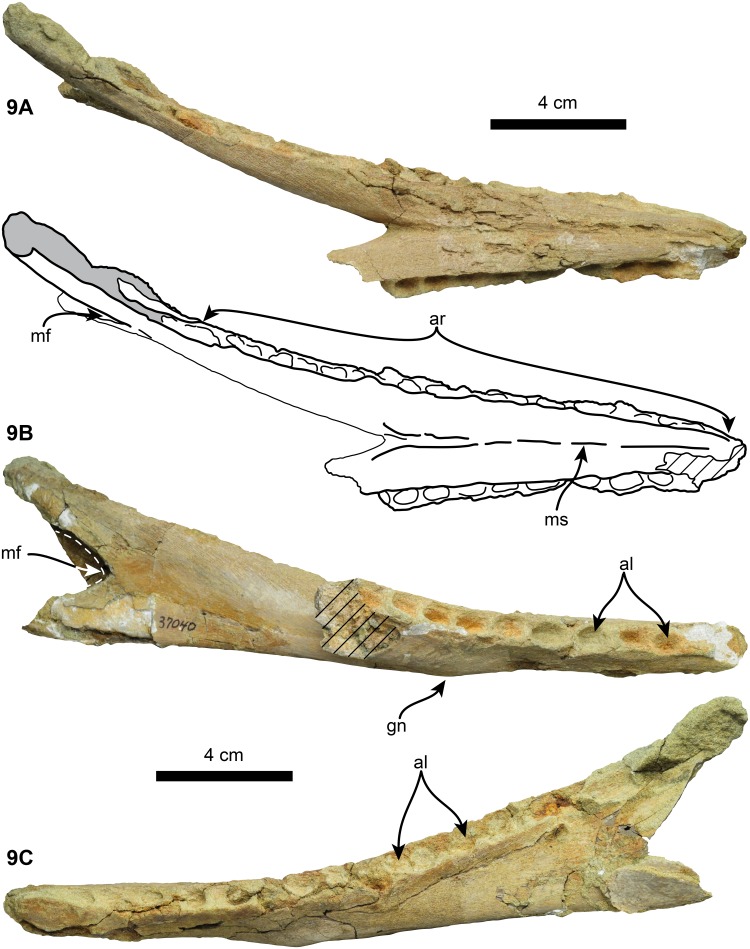
Mandible of holotype (UF 280000) of *Nanokogia isthmia* gen. et sp. nov. in dorsal, 8A, right lateral, 8B, and left lateral 8C, views. Abbreviations: al, tooth alveoli; ar, alveolar row; gn, gnathion; mf, mandibular fossa; ms, mandibular symphysis. Diagonal lines denote broken surfaces.

**Fig 10:**
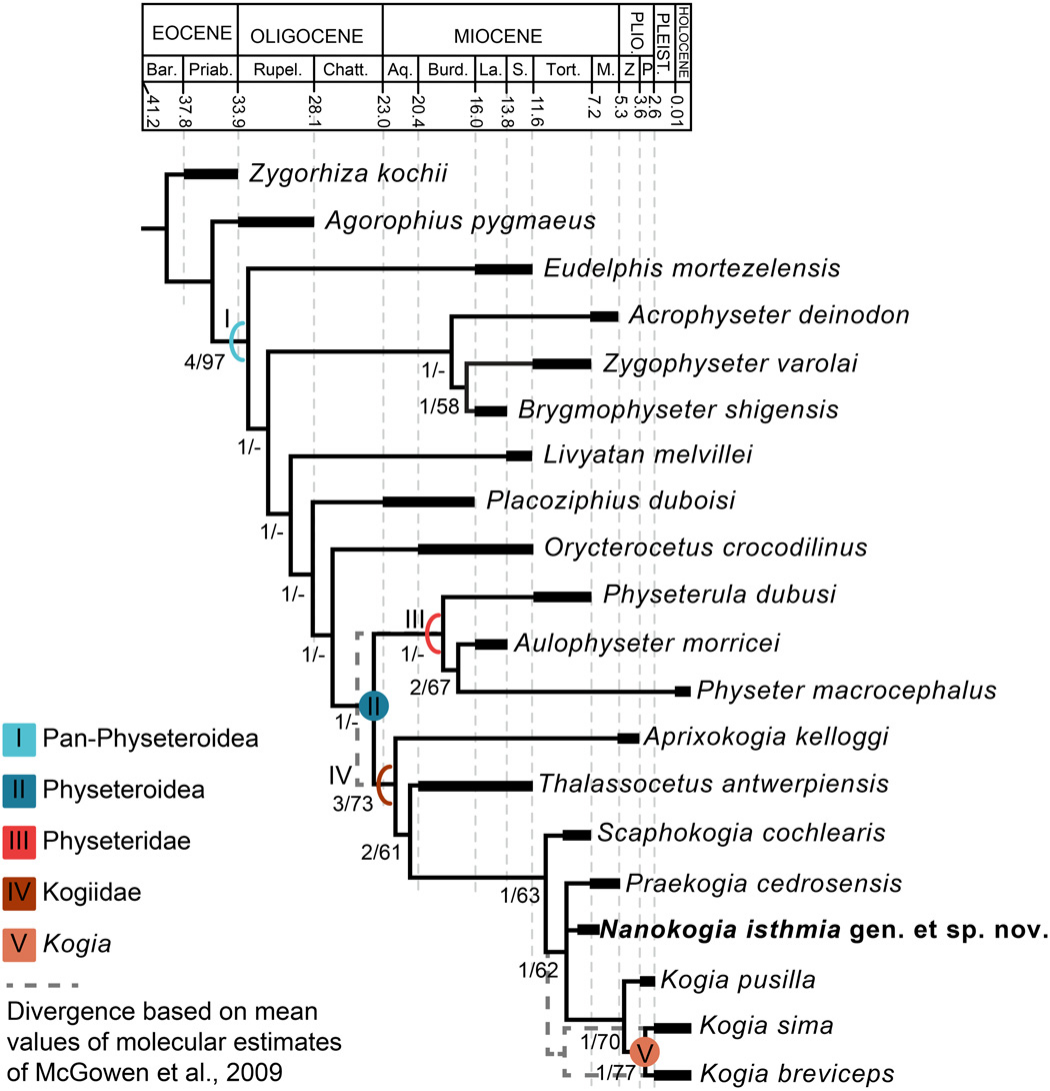
Time-calibrated phylogeny of Pan-Physeteroidea. Strict consensus tree resulting from three most parsimonious trees, 95 steps long, with CI = 0.589 and RI = 0.723. Arcs indicate stem-based taxa, while circles denote node-based clades; numbers below nodes indicate decay indices/bootstrap values; for definition of clades see text. Abbreviations: Aq., Aquitanian; Bar., Bartonian; Burd., Burdigalian; Chatt., Chattian; La., Langhian; M., Messinian; P., Piacenzian; Plio., Pliocene; Pleist., Pleistocene; Priab., Priabonian; Rupel., Rupelian; S., Serravallian; Tort., Tortonian; Z, Zanclean. (Time scale based on Cohen et al. [20].)

**Fig 11:**
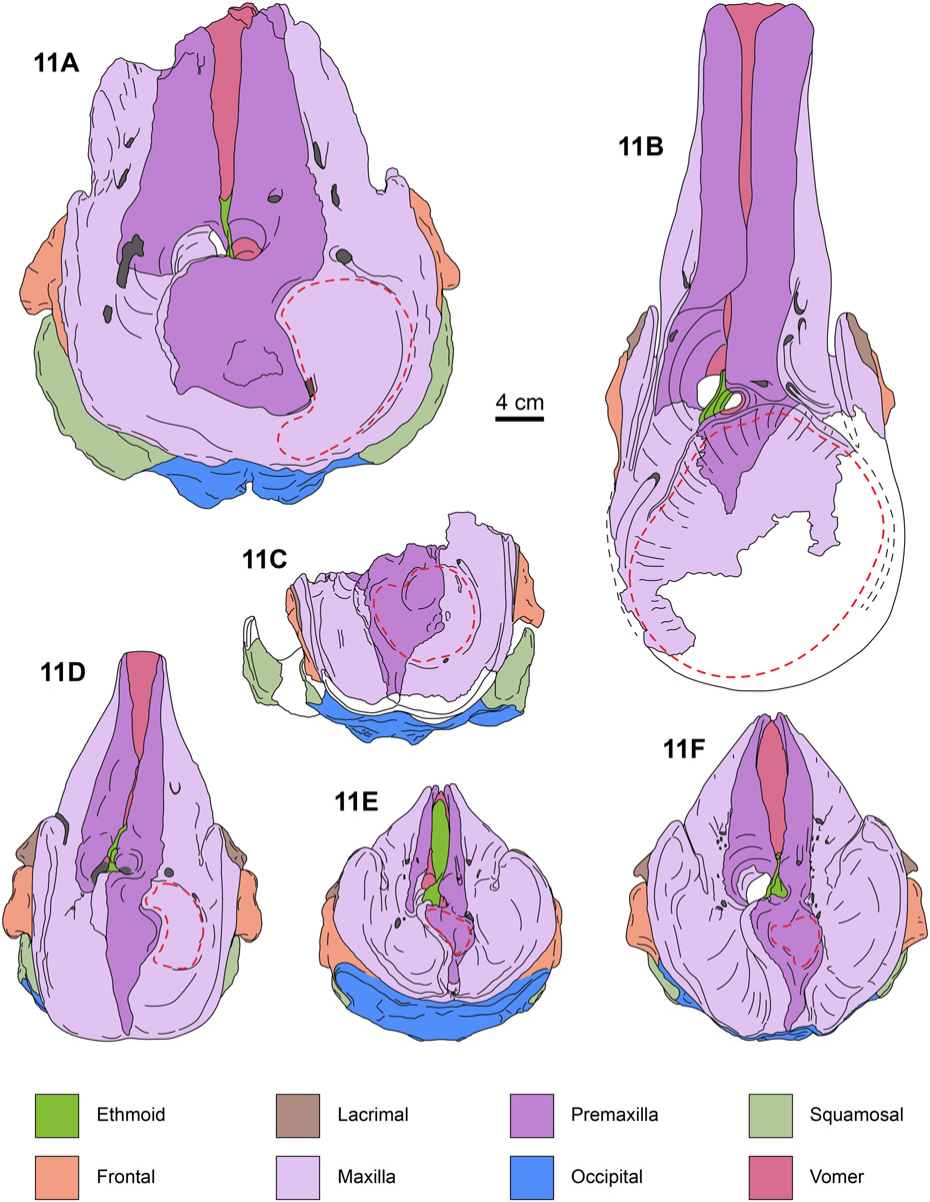
Dorsal views of kogiid skulls. *Aprixokogia kelloggi* (USNM 187015), 11A, *Scaphokogia cochlearis* (MNHN PPI 229), 11B, *Praekogia cedrosensis* (UCMP 315229), 11C, *Nanokogia isthmia* gen. et sp. nov. (based on UF 280000 and 273554), 11D, *Kogia sima* (LACM 47142), 11E, and, *K*. *breviceps* (LACM 95745), 11F. Each bone is color-coded for ease of comparison. Red dashed lines denote the extent of the supracranial/premaxillary fossa. Areas in white are not preserved and have been reconstructed with plaster on the specimens. Illustration of *S*. *cochlearis* modified from Muizon [8]; all other illustrations based on the specimens listed.

**Fig 12:**
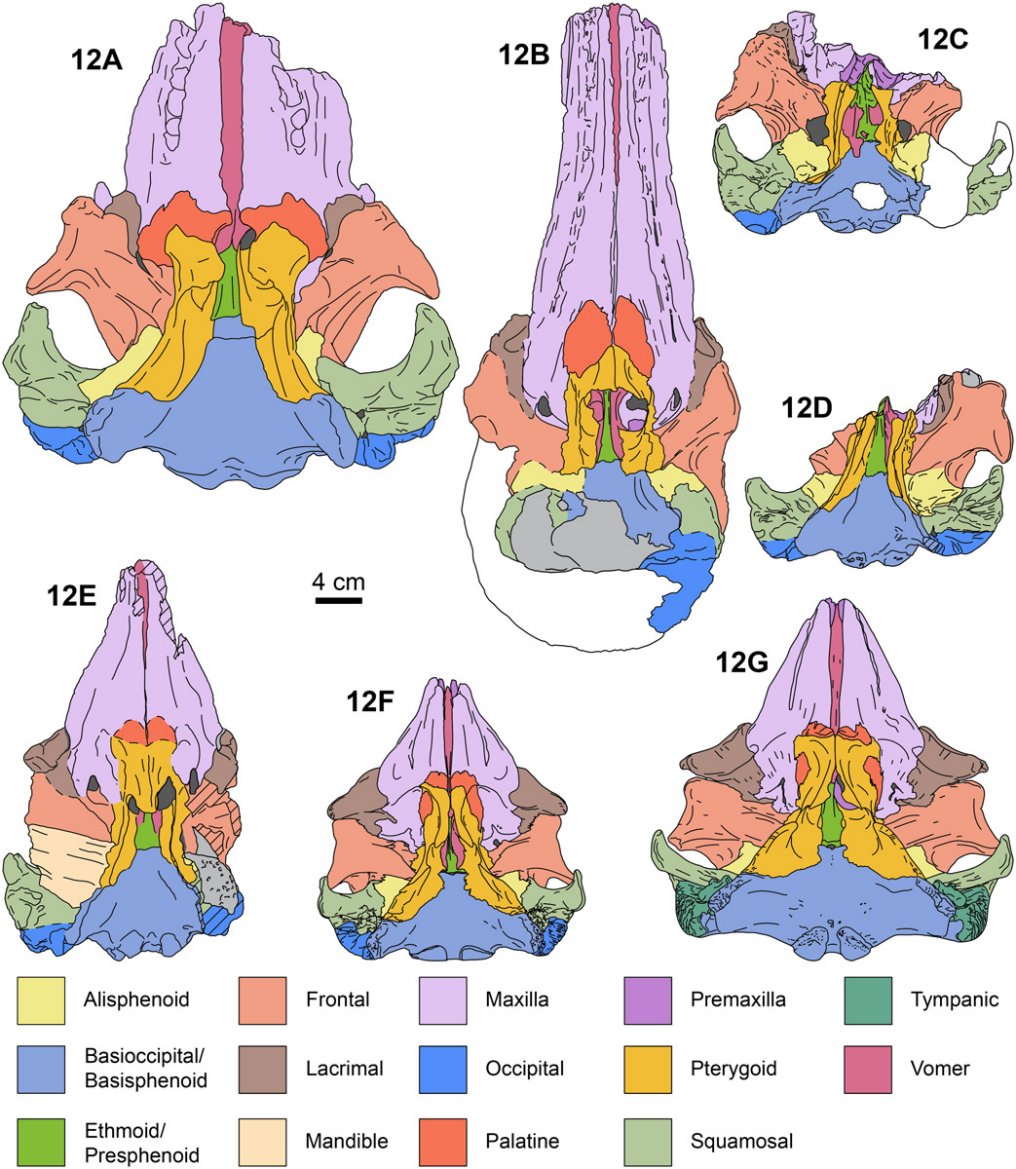
Ventral views of kogiid skulls. *Aprixokogia kelloggi* (USNM 187015), 12A, *Scaphokogia cochlearis* (MNHN PPI 229), 12B, *Praekogia cedrosensis* (UCMP 315229), 12C, *Nanokogia isthmia* gen. et sp. nov. (UF 273554), 12D, and (UF 280000), 12E, *Kogia sima* (LACM 47142), 12F, and *K*. *breviceps* (LACM 95745), 12G. Each bone is color-coded for ease of comparison. Areas in white are reconstructed, light gray areas are covered with sediment; diagonal lines denote broken surfaces. Illustrations based on direct observations of the specimens listed.

**Fig 13:**
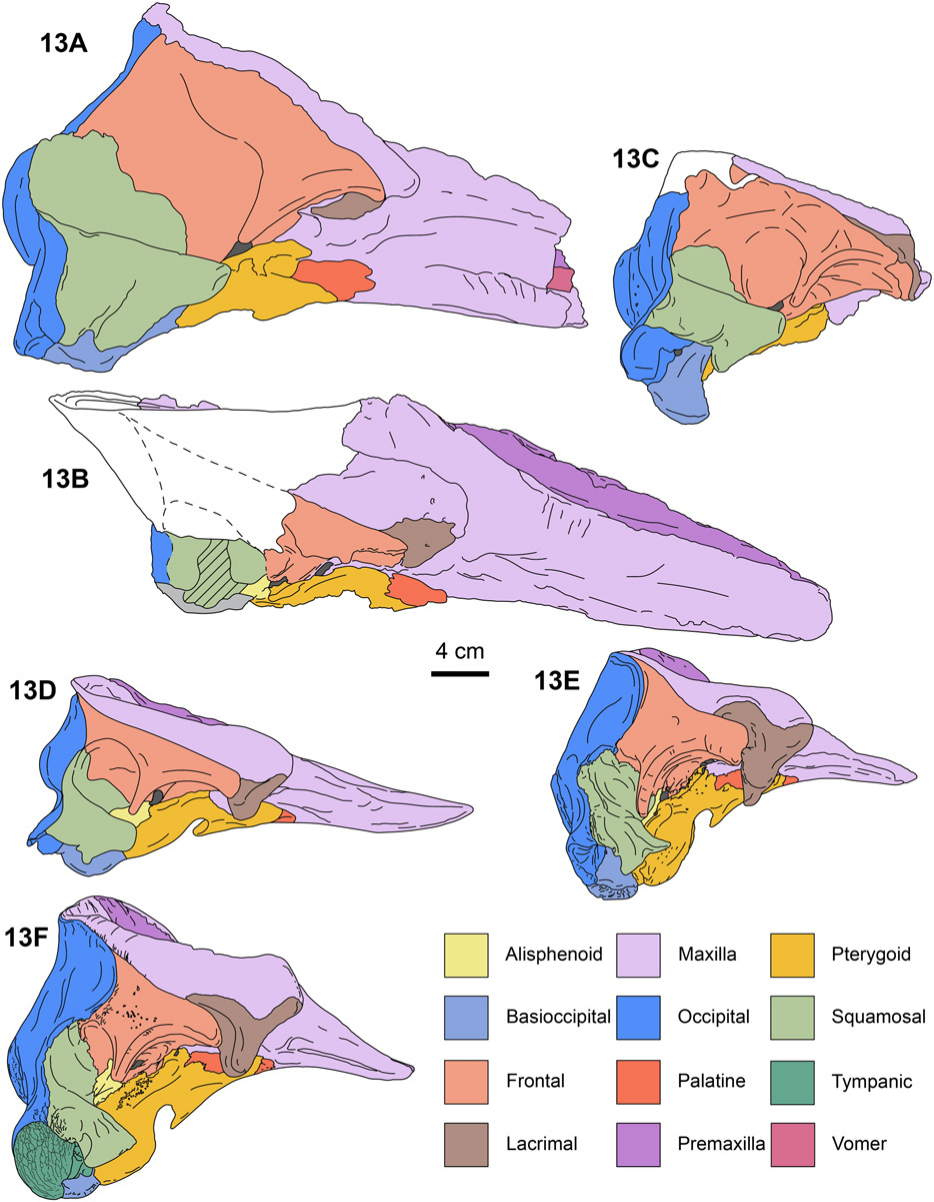
Right lateral views of kogiid skulls. *Aprixokogia kelloggi* (left side, reversed, USNM 187015), 13A, *Scaphokogia cochlearis* (MNHN PPI 229), 13B, *Praekogia cedrosensis* (UCMP 315229), 13C, *Nanokogia isthmia* gen. et sp. nov. (based on UF 280000 and 273554), 13D, *Kogia sima* (LACM 47142), 13E, and *K*. *breviceps* (LACM 95745), 13F. Each bone is color-coded for ease of comparison. Areas in white are reconstructed; diagonal lines denote broken surfaces. Illustrations based on direct observations of the specimens listed.

**Fig 14:**
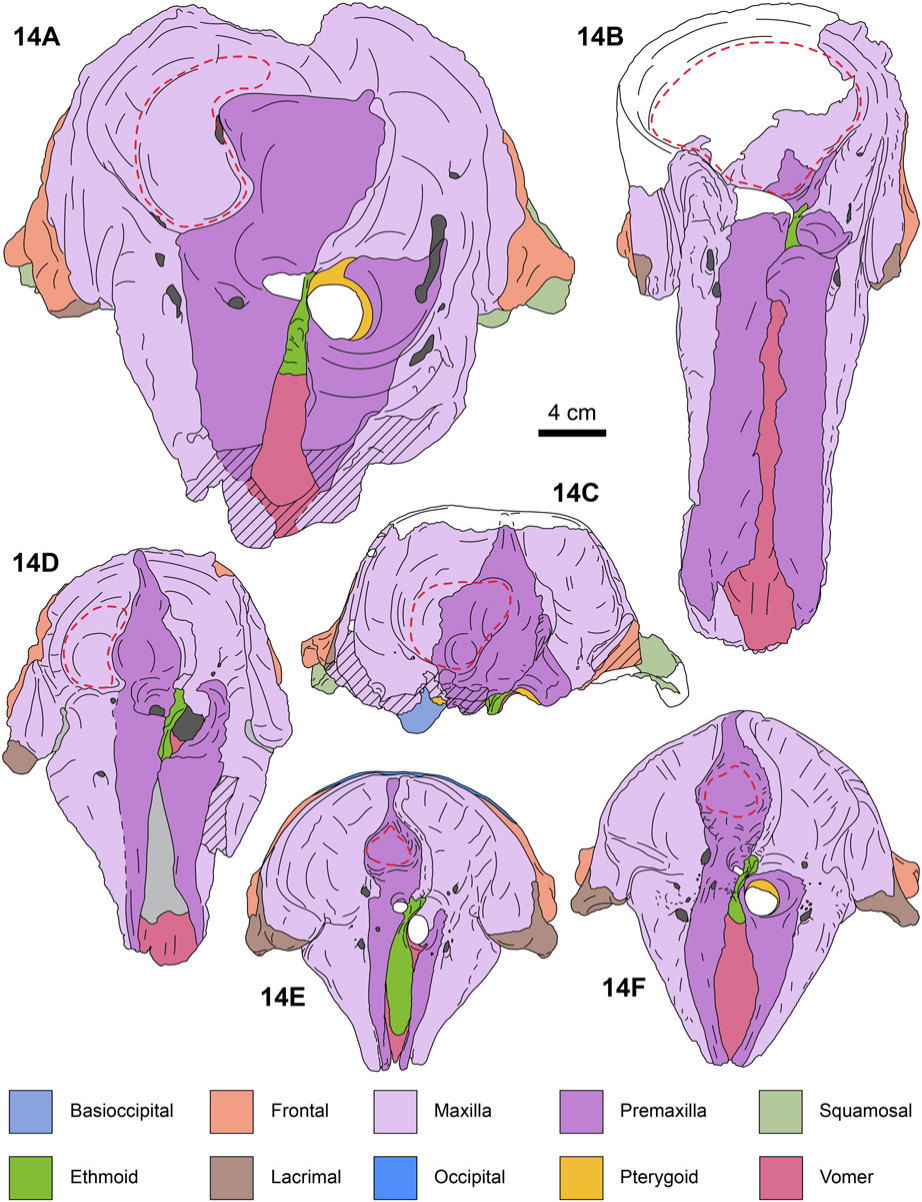
Anterodorsal views of kogiid skulls. *Aprixokogia kelloggi* (USNM 187015), 14A, *Scaphokogia cochlearis* (MNHN PPI 229), 14B, *Praekogia cedrosensis* (UCMP 315229), 14C, *Nanokogia isthmia* gen. et sp. nov. (UF 280000), 14D, *Kogia sima* (LACM 47142), 14E, and *K*. *breviceps* (LACM 95745), 14F; each bone is color-coded for ease of comparison. Red dashed lines denote the extent of the supracranial/premaxillary fossa. Areas in white are reconstructed, light gray areas are covered with sediment; diagonal lines denote broken surfaces.

Description of the skull is based on both the holotype (UF 280000) and the referred specimen (UF 273554) (Figs 2–9, 10–14 and S1–S2 Figs). Because in the temporal and occipital regions the relationships between the different bones are not clear we treat these as separate subdivisions below in the description instead of the individual bones. The holotype is a nearly complete skull, missing parts of the basicranium and parts of the posterolateral surface of the skull. The referred specimen is missing the rostrum, the right supraorbital process and the right half of the supracranial basin. The skull of *Nanokogia* is small (c. 8[0]) (Table 1), asymmetric, with a short rostrum (c. 1[2]) that tapers distally, not gradually as in *Kogia*, and has a marked constriction at about the middle of its length (Figs 2 and 3). The distal end of the rostrum is squared off, not pointed as in *Kogia*, nor cylindrical as in *Scaphokogia*, nor rounded as in *Aprixokogia*. Sutures are fused on both skulls, indicating that they belonged to adult individuals.

#### Premaxilla

The lateral margins of the premaxillae are nearly parallel throughout the length of the rostrum and seem to have reached the anterior tip (c. 2[1]). The dorsal surface along the rostrum is flat to slightly convex. There are no teeth in the premaxillae, as in *Kogia* and *Scaphokogia* (c. 7[1]). There are no foramina on the left premaxilla (c. 15[0]), and none seems to be present on the right one, but this could be due to the poor preservation of the surface. Posteriorly, the left premaxilla curves posteromedially as a tongue-like projection, forming the anterior, lateral and posterior margins of the left naris, thus resembling *Aprixokogia* and *Kogia* spp.; in contrast, the left premaxilla of *Praekogia cedrosensis* is expanded posteriorly, reaching and forming part of the sagittal facial crest [11–12] (Figs 2, 11, 14 and S2 Fig). The right premaxilla forms the anterior, lateral and posterior margins of the right external naris. The external nares are greatly asymmetric as in all pan-physeteroids (c. 18[1]), and the nasals are absent as in all known kogiids (c. 19[2]). The frontal portion of the right premaxilla expands laterally, medially and posteriorly where it joins the left maxilla, together forming the sagittal facial crest (c. 12[1], 14[1]). The dorsal surface of the right premaxilla is flat to convex, thus lacking the central premaxillary fossa seen in *Thalassocetus* and *Kogia* (Lambert [7]:figs 16–17) (Figs 11, 14 and S2 Fig). The lateral edge of the right premaxilla is convex at midlevel of the sagittal facial crest, differing from the concave margin of *Scaphokogia* and *Praekogia*, or the more flange-like margin observed in *Aprixokogia*, *Thalassocetus*, or *Kogia* spp. The posterior half of the sagittal facial crest seems to be composed only of the right premaxilla as in *Praekogia* and differing from *Kogia* where both maxilla and premaxilla form the crest (Figs 2, 11, 14 and S2 Fig). The sagittal facial crest overhangs the left side of the supracranial basin and tapers posteriorly (c. 13[0]), nearly reaching the nuchal crest, similar to the condition in *Praekogia cedrosensis* (Figs 11, 14 and S2 Fig). Overall, the outline of the sagittal facial crest of *Nanokogia* is very similar to what is observed in *Praekogia* and *Kogia*. However, it differs in that the lateral expansion of the right premaxilla at the beginning of the crest is convex, not concave as in *Praekogia*, nor does it overhang laterally over the right side of the supracranial basin as in *Kogia* (Figs 11, 14 and S2 Fig).

#### Maxilla

On the rostrum, the maxilla reaches the distal tip, together with the premaxilla and the vomer (c. 2[1]). When viewed dorsally, the lateral borders of the rostral portion of the maxilla taper anteriorly with a marked constriction at about mid-length, its width diminishing anteriorly and becoming narrower in dorsal view than the premaxilla anteriorly (c. 4[1], 5[0]). Along the rostrum, the dorsal surface of the maxilla is flat to convex, especially in the area anteromedial and medial to the antorbital notches. The antorbital notches are deep, narrow slits (c. 9[2]) entering the supracranial basin (c. 10[1]), as in *Praekogia* and *Kogia*. Posterior to the antorbital notches, the maxillae are greatly expanded over the cranial roof, covering nearly all of the frontals, and forming the supracranial basin (c. 3[1], 21[1]) (Figs 2, 11, 14 and S2 Fig). The outline of the supracranial basin is oval, similar to that of *Praekogia* and contrasting with the more rounded outline of *Kogia*. The lateral maxillary crests are high, with rounded to sharp, medially recurved dorsal margins that slightly overhang the basin, similar to *Praekogia* and differing from the more inflated crests of *Kogia* and the thinner crests of *Aprixokogia*. The lateral maxillary crests of *Nanokogia* reach a maximum height and width of about 20 mm at the level of the antorbital notches; posteriorly the crests diminish in height and thickness (reaching a minimum of ~ 5 mm). Posterolaterally the maxillary crests overhang the temporal fossa and occipital region (Figs 2, 4, 6 and 7). Medially, the left maxilla joins the right premaxilla and forms part of the anterior half of the sagittal facial crest as in *Praekogia* [11] (Figs 2, 11C, 11D, 14C, 14D and S2C–S2D Fig); this condition contrasts with that of *Kogia* where the maxilla forms part of the sagittal facial crest throughout its length [29] (Figs 11E, 11F, 14E, 14F and S2 Fig). On the surface of the supracranial basin, posterolateral to the right external naris, there is a large, kidney-shaped fossa, herein termed supracranial fossa (= premaxillary fossa of Barnes [11]). The deepest part of this fossa is towards its anteromedial edge, where it reaches a maximum depth of about 2 cm; posteriorly it becomes shallower. A similar fossa is present in *Praekogia cedrosensis* and *Aprixokogia kelloggi*. However, in *Praekogia* the fossa is floored by both the premaxilla and maxilla, whereas in *Nanokogia*, as in *Aprixokogia*, it seems to be formed solely by the maxilla. The supracranial fossa of *Aprixokogia* differs from *Nanokogia* and *Praekogia* in that it extends further posteromedially (Figs 11, 14 and S2 Fig). On the right maxilla of *Nanokogia* there are at least three small, dorsal infraorbital foramina (c. 11[0]). The single anterior dorsal infraorbital foramen is located anteromedial to the antorbital notch, is rounded, and has a diameter of about 5 mm. There are also at least two posterior dorsal infraorbital foramina. The largest of these is located posteromedial to the antorbital notch, is rounded, and has a diameter of about 6 mm. The second foramen is located near the anterolateral edge of the supracranial fossa, has a smaller diameter (<5 mm), and continues posterolaterally as a shallow groove along the edge of the fossa. A similar foramen is also observed in *Praekogia cedrosensis* [11] (Figs 2 and 11C). The left maxilla has at least two posterior dorsal infraorbital foramina (Figs 2 and 14D). Both foramina are located posterolateral to the external nares; the anteriormost is the smallest (<5 mm in diameter) and opens anteromedially whereas the second is larger (~7 mm in diameter) and opens dorsally. On the left maxilla of the referred specimen, UF 273554, a single dorsal infraorbital foramen is preserved. It is located posterodorsal to the left external naris, opens laterally, and is anteroposteriorly longer than dorsoventrally wide (~1.5 cm long by 0.5 cm wide (Fig 4A and 4B).

The palatal surface of the maxilla is flat to gently convex. There are no maxillary teeth and only faint indications of a vestigial upper alveolar groove (Figs 3 and 12E; c. 6[1]). The ventrolateral edges are flange-like (maxillary flange of Mead and Fordyce [19]) with their ventral surfaces transversely concave anteromedial to the antorbital notches. The anteriormost extension of the pterygoid sinus is represented by a shallow, oval concave fossa located anteromedial to the ventral infraorbital foramen (Figs 3 and 6), similar to the condition observed in *Kogia* spp. [30]. The infraorbital foramen is located far anteromedial to the frontal groove (Figs 3–5), being at the level of the middle of the supraorbital process of the frontal; the foramen seems to be bounded dorsally and medially by the maxilla, laterally and ventrally by the lacrimal, and posteriorly by the frontal (Figs 3, 4C, 4D and 5).

#### Palatine

The palatines are convex, located medial to the maxillae, but their borders are not well defined (Fig 3).

#### Lacrimal + Jugal

The lacrimal and jugal are fused as in all physeteroids for which these elements are known (c. 22[1]); they are large (~ 5 cm long) and with a triangular outline in lateral view (Fig 5). The ventral tip of this bone is rounded (diameter of ~20 mm) and ends in a blunt tip. In lateral view the posterodorsal corner of the lacrimal + jugal of *Nanokogia* is not deeply wedged between the frontal and maxilla, in contrast to *Scaphokogia*, *Praekogia* and *Kogia* (c. 23[0]) (Muizon [8]:fig. 35; Fig 13). In ventral view the lacrimal contacts the anteromedial surface of the frontal, and its posterior end tapers and curves posteromedially (Fig 4C and 4D).

#### Frontal

With the exception of the supraorbital processes, nearly all of the dorsal surfaces of the frontals are covered by the maxillae (Figs 2, 4, 11D, 14D and S2 Fig). In lateral view the frontal-maxilla suture forms an angle of about 30° relative to the coronal plane (Fig 5; c. 25[1]), similar to most other fossil kogiids but differing from *Kogia* where the angle is greater than 35° (Fig 13; c. 25[2]). The lateral margin of the supraorbital process is oriented parasagittally in its anterior portion, whereas the posterior part, including the postorbital process, is projected posterolateroventrally. The lateral surface between the pre- and postorbital process is laterally concave (Fig 4A and 4B). The preorbital process is at about the same dorsoventral level as the dorsolateral margin of the base of the rostrum (Figs 6 and 13D; c. 24[0]). The postorbital process slightly overhangs the zygomatic process of the squamosal and is separated from the anterodorsal margin of the zygomatic process by about 1 cm (Figs 6 and 13D). The overhang of the postorbital processes is also seen in *Thalassocetus* and more extremely in *Kogia*, whereas there is no overhang in *Aprixokogia* and *Praekogia* (Fig 13). The anterior margin of the preorbital process is rounded and blunt in contrast to the sharper ventral margin of the postorbital process. On the ventral surface of the supraorbital process there is a long (~5.5 cm) frontal groove (Figs 3–5). The groove is oriented anterolaterally at a greater angle than that observed in *Kogia* spp. and leads proximally to the optic foramen (~1 cm in diameter) (Figs 5–7).

#### Temporal Region

The temporal crest is sharp along its posteroventral border; it curves anterodorsally towards the supraorbital processes as a lower, much less prominent crest (Figs 6 and 7). Posterodorsally, the frontal (and/or parietal) and maxilla form a shelf that laterally overhangs the temporal wall and nuchal crest and posteriorly the occipital region to a greater degree than what is observed in *Kogia* spp. and other fossil kogiids (Figs 7 and 12). The temporal fossa is similar to that in *Aprixokogia kelloggi* in being anteroposteriorly elongated in outline in lateral view (c. 26[0]), contrasting with the anteroposteriorly-shortened fossa seen in *Kogia* spp. (Fig 13).

#### Vomer

In dorsal view, the rostral part of the vomer seems to have reached the anterior end of the rostrum and forms the floor and lateral walls of the mesorostral groove (Fig 2). Ventrally, it is exposed along the anterior half of the rostrum as a narrow sliver (Fig 3); however, the sutures are not well preserved. Posteriorly, the vomer is divided into a pair of processes that cover the lateral, but not the ventral, surfaces of the presphenoid (Figs 3, 4C and 4D). This is similar to the condition observed in *Physeter*, *Aprixokogia*, *Scaphokogia*, and *Kogia* spp. [12] but differs from the condition observed in *Praekogia cedrosensis*, where the vomer covers the ventral surface of the bone [11].

#### Ethmoid and Presphenoid

The ethmoid and presphenoid form the bony septum that, along with the vomer, form the medial walls of the internal and external nares. The external nares are greatly asymmetric, with the left naris being more than twice as wide as the right one (c. 18[1]). Posteriorly, the presphenoid widens and contacts the basisphenoid/basioccipital (Figs 3, 4C and 4D).

#### Pterygoid

The pterygoid is long. The hamuli of the right and left pterygoids meet along a midline suture; when viewed ventrally they have a triangular outline. There is a narrow notch with a rounded terminus between the hamular process and the medial lamina as in *Kogia*. The medial lamina (= vaginal processes of Whitmore and Kaltenbach [12]) forms the lateral and posteroventral walls of the internal nares. The pterygoid extends posteriorly as a thin (<5 mm wide) lamina whose ventral edge is inflected medially. Anteriorly, left and right medial laminae are nearly straight and parallel, diverging posterolaterally near the level of the presphenoid-basisphenoid suture, to eventually meet the basioccipital crest (Figs 3, 4C and 4D). The dorsal lamina of the pterygoid is anteroposteriorly shorter (~4 cm long) than the medial lamina ~10 cm), and are oriented perpendicular to each other. The dorsal laminae floor the anteromedial and medial surface of the optic foramen as in *Kogia* spp. (Figs 3, 4C, 4D, 5, 7C and 7D).

#### Alisphenoid

The alisphenoid forms the posteromedial edge of the frontal groove. The ventral surface of the bone is concave, and together with the medial lamina of the pterygoid it forms part of the pterygoid sinus fossa (Fig 4C and 4D). Near the posteromedial border of the bone is the foramen ovale, which is oriented laterally. The foramen is about 7 mm long anteroposteriorly long and 5 mm wide dorsoventrally, and seems to be roofed by the squamosal.

#### Squamosal

The zygomatic process is triangular in cross section (c. 27 [1]). The glenoid fossa is gently concave and oriented anteromedially. The postglenoid process forms the anterior meatal crest and projects farther ventrally than the paroccipital process of the exoccipital in the holotype (c. 28[0]; Fig 6). Medially, the anterior meatal crest, terminates in a short spiny process (Figs 4C, 4D and 5). Posterior to the postglenoid process there is a transverse notch representing the vestigial external auditory meatus (Figs 3, 4C, 4D, 7A and 7B). Medial to the postglenoid process and mandibular fossa is the tympanosquamosal recess (Figs 4 and 5); this area forms a fossa which likely housed the middle ear air sinus as in other kogiids in which this feature is preserved [11]. Further along the posteromedial border of the squamosal is the short (~7 mm high) and ventrally-oriented falciform process (c. 32[2]; Fig 5). The squamosal fossa is mediolaterally concave and slopes anteriorly. Along the dorsolateral border of the zygomatic process, the supramastoid crest is sharp, and joins the temporal crest (= lambdoid crest of Whitmore and Kaltenbach [12]) posteromedially (Fig 7A and 7B). The posteroventral margin of the squamosal is posteriorly concave, with the exoccipital being exposed posterolaterally to a greater degree than is observed in *P*. *cedrosensis* (Fig 7). The posteroventral surface of the squamosal and the anteroventral surface of the exoccipital form an anteroventrally-oriented, deep, round notch (c. 29[1]; Figs 3, 6, 7 and 13), more similar to what is observed in *Praekogia cedrosensis* than to *Kogia* spp. In *Kogia* spp. this notch accommodates the enlarged posterior process of the tympanic (not preserved here), and it is inferred that both *Nanokogia* and *Praekogia* had tympanics with enlarged posterior processes.

#### Basioccipital/Basisphenoid

These two bones are completely fused. The ventral surface is generally flat. The basioccipital crest is long and prominent as in other kogiids (Figs 3, 4C, 4D, 5, 7 and 8).

#### Occipital region

The surface of the occipital region is convex and smooth, forming an angle of about 60° with the long axis of the skull (c. 30[1], 31[1]; Figs 6 and 8). The foramen magnum is roughly circular, and the occipital condyles are dorsoventrally elongated with rugose articular surfaces. The dorsal condyloid fossae are shallow as in *Kogia* and the condyles are separated ventrally by a shallow intercondylar notch. Relative to the occipital condyles, the rostrum is oriented anteroventrally (Fig 13). The paroccipital processes are oriented posterolaterally and are posteriorly concave. The jugular notch is a ~5 mm deep incisure, located between the paroccipital process of the exoccipital and the basioccipital crest. The broken left squamosal and occipital region of the holotype reveal a structure that is interpreted as the cerebral endocast (Fig 3).

### Mandible

Only the mandible of UF 280000 is preserved, although missing parts of the horizontal ramus posterior to the mandibular fossa on the left side and most of the horizontal ramus posterior to the symphysis on the right (Fig 9). The mandibular symphysis is long and fused, with its ventral border straight and keeled (Fig 9, S1 Fig). The alveolar row extends posteriorly to near the anterior margin of the mandibular fossa, and the alveoli are round and oriented dorsolaterally. Posterior to the mandibular symphysis, the lateral alveolar margin overhangs the external surface of the bone. The left ramus preserves alveoli for about 14 small round teeth (c. 37[0], 39[1], 40[1]). Posterior to the symphysis, the medial surface of the horizontal ramus is convex, becoming flatter towards the mandibular foramen. The mandibular fossa is dorsoventrally broad as in other odontocetes, with a V-shaped apex forming its anterior margin. In lateral view, the gnathion marks an abrupt change in the orientation of the ventral surface and the end of the symphysis; the latter is relatively long compared to *Kogia breviceps* and *Kogia sima* (S1 Fig). Ventrally, towards the proximal end of the symphysis there is a pair of oval depressions, which would correspond to the attachment sites of the geniohyoid muscles in *Kogia* [31]. In dorsal view the mandibular rami gradually diverge posterolaterally. The preserved portion of the mandible is about 121 mm long, the symphysis is 101 mm long, and the tooth alveoli are around 10 mm in diameter.

### Phylogenetic Analysis

In order to determine the relationships between *Nanokogia* and other fossil and extant kogiids, we performed a phylogenetic analysis using the character-state matrix for physeteroids of Lambert et al. [21]. The analysis includes two outgroup and 16 ingroup taxa. In contrast to Lambert et al. [21] we treated all characters as unordered, we re-scored some characters for *Livyatan melvillei* based on personal observations of MUSM 1676, *Thalassocetus antwerpiensis* based on the description by Lambert [7], and *Praekogia cedrosensis* based on personal observations of UCMP 315229 (formerly University of California Riverside 15299), and we treated each species of *Kogia* as a separate taxonomic unit (S4 Text). We analyzed the matrix using PAUP* [32], by doing a heuristic search using the tree bisection-reconnection (TBR) algorithm. Statistical support analyses were done by searching for successive longer trees to calculate decay indices and 1000 bootstrap replicates.

The phylogenetic analysis resulted in three most parsimonious trees 95 steps long with consistency index (CI) = 0.589 and retention index (RI) = 0.723. The overall topology of the strict consensus tree is identical to the one shown in Lambert et al. ([21]:Fig 2), with the only difference being the polytomy between *Nanokogia* and *Praekogia* (Fig 10). We use the phylogenetic definitions proposed by Lambert [7] for Physeteridae and Kogiidae. Physeteridae Gray [25] is phylogenetically defined as the group that includes all physeteroids more closely related to *Physeter macrocephalus* Linnaeus [22] than to *Kogia breviceps* (Blainville [1]) and *K*. *sima* (Owen [2]). Kogiidae Gill [26] is defined as the group that includes all physeteroids more closely related to *Kogia breviceps* (Blainville [1]) and *K*. *sima* (Owen [2]) than to *Physeter macrocephalus* Linnaeus [22]. In addition, we phylogenetically define Physeteroidea Gray [25] as the crown group composed of the last common ancestor of *Physeter macrocephalus* Linnaeus [22], *Kogia breviceps* (Blainville [1]), and *K*. *sima* (Owen [2]). Pan-Physeteroidea is defined as the panstem that includes crown Physeteroidea. Finally, *Kogia* Gray [33] is defined as the crown group composed of the last common ancestor of *Kogia breviceps* (Blainville [1]) and *K*. *sima* (Owen [2]), and all its descendants.

Additionally, we performed an analysis of the matrix using the same parameters outlined above but setting certain characters as ordered, following Lambert et al. [21]. This analysis resulted in 73 most parsimonious trees 102 steps long with consistency index (CI) = 0.569 and retention index (RI) = 0.723. The resulting strict consensus tree (S3 Fig) included an unresolved polytomy between crownward Pan-Physeteroidea, Physeteridae and Kogiidae, with the *Acrophyseter* + *Zygophyseter* + *Brygmophyseter* clade fully collapsed, and *Physeterula* no longer being within Physeteridae. Kogiidae remained largely stable, only with a polytomy between *Praekogia* and *Nanokogia*.

## Discussion

### Comparison and Relationships

*Nanokogia isthmia* is a derived kogiid most closely related to *P*. *cedrosensis* and *Kogia* spp. (Fig 10). Among these, the overall morphology of *Nanokogia* resembles *Praekogia cedrosensis* much more than any of the other taxa, especially in the shape of the supracranial basin and its small size (Figs 11–14 and S2 Fig). In contrast, the differences in cranial morphology between *Nanokogia* and *Scaphokogia*, both from Tortonian-age deposits, are striking; a greater degree of cranial disparity is observed between them compared to the disparity observed between *Kogia breviceps* and *K*. *sima* (Figs 11–14 and S2 Fig). In fact, both extant species of *Kogia* are so similar that, until 1966, they were considered to represent a single species [5].

The rostrum of *Nanokogia* resembles more closely that of *Kogia* spp. by having a triangular outline in dorsal and ventral views, contrasting with the more rounded outline of the rostrum of *Aprixokogia* and the cylindrical outline of *Scaphokogia* (Figs 11–14 and S2 Fig). However, the dorsal surface of the rostrum of *Nanokogia* is not as concave as that of *Kogia* or *Physeter*, resembling in that respect the more flattened to shallowly convex dorsal surface of the rostrum of *Aprixokogia*, while the markedly convex rostrum of *Scaphokogia* is unique among kogiids (Fig 14, S2 Fig). In lateral view, the height of the rostrum is fairly similar in *Nanokogia*, *Aprixokogia* and *Kogia*, while in *Scaphokogia* it is notably greater, giving it a cylindrical outline (Figs 11–14 and S2 Fig). The concave dorsal surface of the rostrum of *Kogia*, *Physeter* and *Livyatan* [21] can be considered as an extension of the supracranial basin, and accommodates the hypertrophied soft tissue structures of the forehead, namely the melon in the former, and junk and spermaceti organ in the latter two. These structures project anterodorsally beyond the limits of the rostrum (Cranford et al. [34]:Fig 8; Cranford [35]:Fig 3). The flat to concave dorsal surface of the rostrum of *Nanokogia*, *Aprixokogia* and *Scaphokogia* suggests that the associated soft tissue was less hypertrophied anteriorly in these taxa.

The basicranium of *Nanokogia isthmia* shows a unique combination of morphological features that sets it apart from other kogiids. *Nanokogia* differs from *P*. *cedrosensis* in that the presphenoid is not covered ventrally by the vomer, a characteristic which *Nanokogia* shares with *Physeter macrocephalus*, *Aprixokogia kelloggi*, *Scaphokogia cochlearis*, and *Kogia* spp. [12, 29] (Fig 12). However, it does share with *Praekogia* and *Kogia* spp. the presence of a wide notch in the squamosal for the posterior process of the tympanic bulla; this notch is much shallower in *Aprixokogia*, and absent in *Thalassocetus* [7, 12] (Fig 13). *Nanokogia* resembles *Aprixokogia* in having a temporal fossa that is longer than high; thus it differs from the rounded fossa of *Praekogia*, and from *Thalassocetus* and *Kogia* spp. where the temporal fossa is higher than long.

The pterygoid of *Nanokogia* resembles that of *Kogia* in the presence of a notch between the hamular process and the medial lamina (Fig 13D–13F); this feature is either absent or not preserved in other kogiids. However, in lateral view, the pterygoid medial lamina and other features of the basicranium of *Kogia* are oriented more anteroventrally than in *Aprixokogia*, *Nanokogia*, or, probably, *Scaphokogia*. Based on the shape of the basioccipital crest, *Praekogia* seems to be similar to *Kogia* (Fig 13). Because of this reorientation, the basicranium of *Kogia* looks anteroposteriorly shortened or recurved in comparison to other kogiids (Fig 13: i.e., notice the more vertical or diagonal orientation of the long axis of the pterygoid in *Kogia*). This results in the postorbital process of the frontal overhanging the posterior half of the zygomatic process, foreshortening of the temporal fossa, and reposition of the glenoid fossa to a more ventral position relative to the rostrum (Fig 13E and 13F), likely having an effect on the shape of the mandible as well.

*Nanokogia* is the only fossil kogiid for which an associated mandible is known. The mandible of *Nanokogia* differs from that of *Kogia breviceps* and *K*. *sima* in having a straight ventral border of the horizontal ramus (Fig 9 and S1 Fig). In addition, the orientation of the mandible posterior to the gnathion differs markedly between *Nanokogia* and *Kogia*: the orientation in the former is posterodorsal (S1A Fig), while in the latter it is posteroventral (S1B–S1C Fig), with the mandibular condyle located far ventrally with respect to the alveolar row. These differences are most likely the result of the reorientation of the basicranium of *Kogia* as mentioned above.

The supracranial basin also shows marked differences among kogiids (Figs 11–14 and S2 Fig). In nearly all taxa, the supracranial basin is anterodorsally oriented as the posterior edge of the basin is elevated, although not as much as in pan-physeteroids and physeterids (e.g. *Acrophyseter deinodon* [36] and *Physeter macrocephalus* [37]); the one exception being *Scaphokogia* whose basin seems to be oriented dorsally (Fig 13). The sagittal facial crests of *Nanokogia*, *Aprixokogia*, *Scaphokogia*, and *Kogia* spp. differs from that in *Praekogia* in that the left premaxilla does not reach and form part of the sagittal facial crest (Figs 11, 14 and S2 Fig). The sagittal facial crest of *Aprixokogia* differs from that of other kogiids in that it does not taper posteriorly and does not reach the posterior margin of the supracranial basin (Fig 14). The crest is tapering and proportionately longer in *Thalassocetus*, *Nanokogia*, *Praekogia* and *Kogia*, but is much reduced in *Scaphokogia*. Both *Nanokogia* and *Praekogia* share with *Aprixokogia* the presence of a supracranial fossa mostly confined to the right side of the supracranial basin, although the extent of the fossa differs slightly (see Description) (Figs 11, 14 and S2 Fig). In *Scaphokogia cochlearis* this fossa is developed to an extreme, with the sagittal facial crest displaced towards the left side of the supracranial basin, resulting in a much larger fossa [8] (Figs 11, 14 and S2 Fig). The supracranial fossa of these taxa seems to be homologous to the central premaxillary fossa of *Kogia* spp. and *Thalassocetus* ([7, 14]; but see [11] for a different interpretation). In *Kogia*, the central premaxillary fossa corresponds to the nasofrontal sac and the relatively small spermaceti organ [34, 38–39]. Muizon [8] and Bianucci and Landini [14] interpreted the enlarged fossa of *Scaphokogia* as indicative of an enlarged spermaceti organ. Considering these two structures, the supracranial fossa and central premaxillary fossa, as homologous, we hypothesize that *Aprixokogia*, *Praekogia*, and *Nanokogia* also possessed a spermaceti organ proportionately larger than that of *Thalassocetus* and *Kogia* spp., which will also be the most parsimonious explanation, according to our phylogenetic analysis (see below).

The results of our phylogenetic analysis place *Aprixokogia kelloggi* as the most basal kogiid (Fig 10). This, together with the presence of an enlarged supracranial basin in pan-physeteroids (e.g. *Acrophyseter deinodon* [36], *Zygophyster varolai* [40]), indicates that an enlarged spermaceti organ is most likely the plesiomorphic condition for crown physeteroids. This suggests that reduction of the spermaceti organ has evolved iteratively within kogiids: once in *Thalassocetus antwerpiensis*, and secondly in *Kogia* spp. In *Physeter macrocephalus* the spermaceti organ is related mainly to sound generation [39–40], but has also been considered as used for interspecific aggression [41], among other functions. In *Kogia* it seems that reduction of the organ has resulted in changes in its functionality relative to *Physeter*, and it has become part of a complex sound-generating system [39]. It has been hypothesized that strong sexual selection has influenced the hypertrophy of the spermaceti organ and nose of *Physeter* [34], so it could be argued that the smaller organ of *Kogia* is the result of less intense sexual selection. Furthermore, cetaceans that display strong sexual selection tend to have larger pelvic bones [42], which in *Kogia* seem to be extremely reduced or absent [43]. However, we would need more data from living kogiids as well as fossils with associated cranial and postcranial material in order to test this further.

Our results also differ from previous analyses regarding previously published divergence estimates between *Kogia breviceps* and *K*. *sima*. McGowen et al. [44] estimated that the average time of divergence between *Kogia breviceps* and *K*. *sima* was around 9.33 Ma (based on a 4.03–15.38 Ma range). Instead, our results, which place *Kogia pusilla* from the late Pliocene of Italy [14] as the fossil taxon most closely related to extant *Kogia*, coupled with the occurrence of *Kogia*-like periotics in the early Pliocene Yorktown Formation of North Carolina ([45]; JVJ pers. obs.), imply that the divergence between extant *Kogia* occurred sometime after the early Pliocene, and that it was the result of a much more recent speciation event (Fig 14).

### Paleoecology

The depositional environment of the Chagres Formation has been a subject of debate, with a variety of paleobathymetric depths estimated for its three distinct members [16–17]. The Piña facies, from which *Nanokogia* was collected, has been widely described as upper bathyal following Collins et al. ([16]:Fig 1). De Gracia et al. [46] used fossil osteichthyan and chondrichthyan occurrences to characterize the Piña facies as reflecting both neritic and mesopelagic open-ocean settings, giving a paleobathymetric estimate of 100–700 m. More recently, Hendy et al. [17], using the mollusk assemblage, estimated the depositional depth of the Piña facies to be typical of the outer continental shelf, around 100–150 m of water depth. The fish assemblage, which includes billfishes [47] and abundant myctophids [48], among others, is suggestive of areas of coastal upwelling [46]. This upwelling system likely supported a large diversity of fishes and pelagic invertebrates, such as squids.

*Kogia breviceps* and *K*. *sima* feed mainly on squids (teutophagy), but are also known to consume myctophids [49–50], feeding mainly on prey within the epi- and mesopelagic zones [5, 51]. Because of the morphological similarities between the rostrum and other features of the skull of *Nanokogia* and that of *Kogia*, coupled with other paleontological and geological evidence (i.e. high abundance of myctophids, presence of teuthid statoliths, and paleobathymetry estimates for the Piña facies), we hypothesize that *Nanokogia* fed mainly on diel-migrating fishes (e.g. myctophids) and squids; while the relatively short rostrum is indicative of suction feeding [52]. However, *Nanokogia* may have had different sound-generating capabilities, as its spermaceti organ was likely larger than that of *Kogia* (see Comparison and Relationships). Furthermore, when we compare *Nanokogia* with the other known Tortonian kogiid, *Scaphokogia cochlearis*, it is evident that there is a greater morphological disparity of the rostrum between these coeval taxa than what is observed between modern species (Figs 11–13 and S2 Fig). The disparity between *Nanokogia* and *Scaphokogia* is probably related to different feeding or foraging specializations in the latter. Unfortunately there are no extant analogs for the unique cranial morphology of *Scaphokogia*, making it difficult to infer its paleoecology.

### Fossil Marine Mammals of Central America

There are few other reports of fossil marine mammals from Central America. These include a balaenopterid from the lower Pliocene of Nicaragua [53], odontocetes from the mid- and upper Miocene of Costa Rica [54–55], and sirenians and odontocetes from the lower through upper Miocene of Panama [56–58]. Based on isolated teeth, several odontocete families were tentatively identified from the Miocene of Costa Rica [54–55]. However, because isolated teeth are poorly diagnostic and highly convergent amongst very different groups, these identifications must be viewed with caution. Previously described marine mammals from Panama consist of isolated postcranial elements (ribs and vertebrae) representing unknown sirenians, mysticetes and odontocetes, from other coeval or older formations [56–57]. So far the only other odontocete reported from the Chagres Fm. is an isolated tooth of a pan-physeteroid. The tooth was described by Vigil and Laurito [58] as a physeterid, although it should be more properly referred to as a pan-physeteroid due to the presence of an enameled crown. Additional cetacean material from the Piña facies, under study by one of us (JVJ), includes an undescribed pan-physeteroid with *Scaldicetus*-type teeth (teeth with large bulbous roots and small enameled crowns; Smithsonian Tropical Research Institute [STRI] 34111), an inioid (USNM 546125), and remains of small delphinoids (STRI 37037 and STRI 37039), hinting at a greater diversity of late Miocene Central American marine mammals than currently known. This would be consistent with the diversity seen in other contemporaneous or nearly contemporaneous sites in North America (e.g. Agricola [59] and Isla Cedros [60–61] faunas in Florida and Baja California, respectively) and South America (e.g. Montemar [10] and Cerro Ballena [62] faunas in Peru and Chile, respectively).

## Conclusions

Herein we described *Nanokogia isthmia*, from late Miocene (Tortonian) deposits on the Caribbean coast of Panama, and analyzed disparity and temporal patterns among kogiids, including the living dwarf and pygmy sperm whales. A phylogenetic analysis places *Nanokogia* in a polytomy with *Praekogia cedrosensis*, more closely related to *Kogia* spp., than to other taxa. However, *Nanokogia* shows a unique combination of morphological features that set it apart from these and other kogiids. Based on morphological features that *Nanokogia* shares with *Kogia*, as well as other lines of evidence from the fossil record, we hypothesized that the Panamanian kogiid fed mainly on diel-migrating fishes and squids by suction feeding. Based on our phylogenetic analysis, we concluded that an enlarged spermaceti organ is the plesiomorphic condition for kogiids, and that reduction of the organ has occurred iteratively, at least twice within the clade. Furthermore, the crown clade *Kogia* seems to have originated at or after the early Pliocene, which is later than previously published molecular estimates. Accordingly, we suggest that future studies of cetacean phylogeny take more into account the use of fossils for calibrations of their trees at all possible taxonomic levels.

In addition to *Nanokogia isthmia*, other cetaceans from the Piña facies of the Chagres Fm. include physeteroids, inioids, and small delphinoids, indicating that the upper Miocene of Panama may hold a diverse array of marine mammals similar to other contemporaneous sites in North and South America. Finally, the presence of kogiids in the Neotropics shows that the group has been present in the region at least since the late Miocene, and highlights the importance of research in this region in order to further understand the evolutionary history of marine mammals.

## Supporting Information

S1 Dataset List of characters and states, and matrix used in the phylogenetic analysis. (DOC)

S1 Fig Lateral views of kogiid mandibles. Lateral views of mandibles of *Nanokogia isthmia* gen. et sp. nov. (UF 280000) S1A, *Kogia sima* (right side, reversed, LACM 47142), S1B, and *Kogia breviceps* (LACM 95745), S1C. Dashed lines denote the ventral curvature of the symphysis. Abbreviations: gn, gnathion. Scale applies to all specimens.
(TIF)

S2 Fig Anterodorsal views of kogiid skulls. Anterodorsal views of skulls of *Aprixokogia kelloggi* (LACM 117744 [cast of USNM 187015]), S2A, *Scaphokogia cochlearis* (MNHN PPI 229), S2B, *Praekogia cedrosensis* (UCMP 315229), S2C, *Nanokogia isthmia* gen. et sp. nov. (UF 280000), S2D, *Kogia sima* (LACM 47142), S2E, and *Kogia breviceps* (LACM 95745).
(TIF)

S3 Fig Strict consensus tree. Strict consensus tree out of 73 most parsimonious trees obtained from a phylogenetic analysis where certain characters were ordered following Lambert et al. [21].
(TIF)

## References

[1] Blainville H de. Sur les cachalots. Ann Fr Étrang d’Anat Physiol. 1838; 2: 335–337.

[2] Owen R. On some Indian Cetacea collected by Walter Elliot, Esq. Trans Zool Soc London. 1866; 6:17–47.

[3] Willis PM, Baird RW. Status of the dwarf sperm whale, *Kogia simus*, with special reference to Canada. Can Field Nat. 1998; 112: 114–125.

[4] Bloodworth BE, Odell DK. *Kogia breviceps* (Cetacea: Kogiidae). Mammalian Species. 2008; 819: 1–12.

[5] McAlpine D. Pygmy and dwarf sperm whales In: Perrin WF, Würsig B, Thewissen JGM, editors. Encyclopedia of marine mammals. San Diego: Academic Press; 2002 pp. 1007–1009.

[6] Abel O. Les odontocètes du Boldérien (Miocène supérieur) des environs d’Anvers. Mém Mus Royal Hist Nat Belgique. 1905; 3: 1–155.

[7] Lambert O. Sperm whales from the Miocene of the North Sea: a re-appraisal. Bull Inst R Sc N B-S. 2008; 78: 277–316.

[8] de Muizon C. Les vertébrés fossiles de la Formation Pisco (Pérou). Troisième partie: Les odontocètes (Cetacea, Mammalia) du Miocène. Travaux de l’Institut Français d’Etudes Andines. 1988; 78: 1–244. 10.1179/acb.2002.068 17356899

[9] Ehret DJ, MacFadden BJ, Jones DS, DeVries TJ, Foster DA, Salas-Gismondi R. Origin of the white shark *Carcharodon* (Lamniformes: Lamnidae) based on recalibration of the upper Neogene Pisco Formation of Peru. Palaeontology. 2012; 55: 1139–1153.

[10] Lambert O, de Muizon C. A new long-snouted species of the Miocene pontoporiid dolphin *Brachydelphis* and a review of the Mio-Pliocene marine mammal levels in the Sacaco Basin, Peru. J Vertebr Paleontol. 2013; 33: 709–721.

[11] Barnes LG. *Praekogia cedrosensis*, a new genus and species of fossil pygmy sperm whale from Isla Cedros, Baja California, Mexico. Contrib Sci, Nat Hist Mus Los Angeles County. 1973; 247: 1–20.

[12] Whitmore FC Jr, Kaltenbach JA. Neogene Cetacea of the Lee Creek Phosphate Mine, North Carolina; In: Ray CE, Bohaska DJ, Koretsky IA, Ward LW, Barnes LG, editors. Geology and Paleontology of the Lee Creek Mine, North Carolina, IV Virginia Mus Nat Hist S Pub 14; 2008 pp. 181–269.

[13] Pilleri G. The Cetacea of the Italian Pliocene with a descriptive catalogue of the species in the Florence Museum of Paleontology. Vammala: Vammalan Kirjapaino Oy; 1987.

[14] Bianucci G, Landini W. *Kogia pusilla* from the middle Pliocene of Tuscany (Italy) and a phylogenetic analysis of the family Kogiidae (Odontoceti, Cetacea). Riv Ital Paleontol S. 1999; 105: 445–453.

[15] Fitzgerald EMG. Pliocene marine mammals from the Whalers Bluff Formation of Portland, Victoria, Australia. Mem Mus Victoria. 2005; 62: 67–89.

[16] Collins LS, Coates AG, Berggren WA, Aubry MP, Zhang J. The late Miocene Panama isthmian strait. Geology. 1996; 24: 687–690.

[17] Hendy A JW, Jones D, De Gracia C, Velez-Juarbe J. Paleoecology of the Chagres Formation (latest Miocene) of Panama: reinterpreting the paleoenvironment of a vertebrate-rich marine fauna. J Geol. In review.

[18] Perrin WF. Variation of spotted and spinner porpoise (genus *Stenella*) in the eastern Pacific and Hawaii. Bull Scripps Inst Oceanog Univ Calif. 1975; 21: 1–206.

[19] Mead JG, and Fordyce RE. The therian skull: a lexicon with emphasis on the odontocetes. Sm C Zool. 2009; 627: 1–248.

[20] Cohen KM, Finney SC, Gibbard PL, Fan J-X. The ICS international chronostratigraphic chart. Episodes. 2013; 36: 199–204.

[21] Lambert O, Bianucci G, Post K, de Muizon C, Salas-Gismondi R, Urbina M, et al The giant bite of a new raptorial sperm whale from the Miocene epoch of Peru. Nature. 2010; 466: 105–108. 10.1038/nature09067 20596020

[22] Linnaeus C. Systema naturae per regna tria naturae, secundum classes, ordines, genera, species, cum characteribus, differentiis, synonymis, locis Tomus 1, Editio decima, reformata. Stockholm: Laurentii Salvii; 1758.

[23] Brisson MJ. Regnum animale in Classes IX distributum, sive synopsis methodica sistens generalem animalium distributionem in Classes IX, et duarum primarum Classium, Quadrupedum scilicet & Cetaceorum, particulare divisionem in Ordines, Sectiones, Genera, et Species. Paris: T. Haak; 1762.

[24] Flower WH. Description of the skeleton of *Inia geoffrensis* and of the skull of *Pontoporia blainvilii*, with remarks on the systematic position of these animals in the order Cetacea. Trans Zool Soc London. 1867; 6: 87–116.

[25] Gray JE. On the natural arrangement of vertebrose animals. London Med Rep. 1821; 15: 296–310.

[26] Gill T. The sperm whales, giant and pygmy. Am Nat. 1871; 4: 725–743.

[27] Pyenson ND, Sponberg SN. Reconstructing body size in extinct crown Cetacea (Neoceti) using allometry, phylogenetic methods and tests from the fossil record. J Mamm Evol. 2011; 18: 269–288.

[28] Caldwell DK, Caldwell MC. Pygmy sperm whale *Kogia breviceps* (de Blainville, 1838): Dwarf sperm whale *Kogia simus* Owen, 1866 In: Ridgway SH, Harrison R, editors. Handbook of Marine Mammals, vol. 4: River dolphins and the larger toothed whales. London: Academic Press; 1989 pp. 235–260.

[29] Schulte HvonW. The skull of *Kogia breviceps* Blainv. Bull Am Mus Nat Hist. 1917; 37: 361–404.

[30] Fraser FC, Purves PE. Hearing in cetaceans. Evolution of the accessory air sacs and the structure and function of the outer and middle ear in recent cetaceans. Bull British Mus Nat Hist, Zool. 1960; 7: 1–140.

[31] Schulte HvonW, Smith MDeF. The external characters, skeletal muscles, and peripheral nerves of *Kogia breviceps* (Blainville). Bull Am Mus Nat Hist. 1918; 38: 7–72.

[32] Swofford DL. PAUP* v.40b10. Sinauer Associates, Sunderland; 2002.

[33] Gray JE. On the cetaceous animals In: Richardson J, Gray JW, editors. The zoology of the voyage of *H*. *M*. *S*. *Erebus* and *Terror* under the command of Captain Sir James Clark Ross, R. N., F. R. S., during the years 1839 to 1843. Vol. 1, pt. 3 (Mammals). London: Longman, Brown, Green and Longmans; 1846 pp. 13–53.

[34] Cranford TW, Amundin M, Norris KS. Functional morphology and homology in the Odontocete nasal complex: implications for sound generation. J Morphol. 1996; 228: 223–285. 8622183 10.1002/(SICI)1097-4687(199606)228:3<223::AID-JMOR1>3.0.CO;2-3

[35] Cranford TW. The sperm whale’s nose: sexual selection on a grand scale? Mar Mamm Sci. 1999; 15: 1133–1157.

[36] Lambert O, Bianucci G, de Muizon C. A new stem-sperm whale (Cetacea, Odontoceti, Physeteroidea) from the latest Miocene of Peru. C R Palevol. 2008; 7: 361–369.

[37] Huggenberger S, André M, Oelschläger HHA. The nose of the sperm whale: overviews of functional design, structural homologies and evolution. J Mar Biol Assoc UK. 2014.

[38] Kernan JD Jr, Schulte HvonW. Memoranda upon the anatomy of the respiratory tract, foregut, and thoracic viscera of a fetal *Kogia breviceps* . B Am Mus Nat Hist. 1918; 38: 231–266.

[39] Clarke MR. Production and control of sound by the small sperm whales, *Kogia breviceps* and *K*. *sima* and their implications for other Cetacea. J Mar Biol Assoc UK. 2003; 83: 241–263.

[40] Bianucci G, Landini W. Killer sperm whale: a new basal physeteroid (Mammalia, Cetacea) from the Late Miocene of Italy. Zool J Linn Soc-Lond. 2006; 148: 103–131.

[41] Carrier DR, Deban SM, Otterstrom J. The face that sank the *Essex*: potential function of the spermaceti organ in aggression. J Exp Biol. 2002; 205: 1755–1763. 12042334 10.1242/jeb.205.12.1755

[42] Dines JP, Otárola-Castillo E, Ralph P, Alas J, Smith AD, Dean MD. Sexual selection targets cetacean pelvic bones. Evolution. 2014; 68: 3296–3306. 10.1111/evo.12516 25186496 PMC4213350

[43] Benham WB. On the anatomy of *Cogia breviceps* . Proc Zool Soc London. 1901; 71: 107–135.

[44] McGowen MR, Spaulding M, Gatesy J. Divergence date estimation and a comprehensive molecular tree of extant cetaceans. Mol Phylogenet Evol. 2009; 53: 891–906. 10.1016/j.ympev.2009.08.018 19699809

[45] Luo Z, Marsh K. Petrosal (periotic) and inner ear of a Pliocene kogiine whale (Kogiinae, Odontoceti): implications on relationships and hearing evolution of toothed whales. J Vertebr Paleontol. 1996; 16: 328–348.

[46] De Gracia C, Carrillo-Briceño J, Schwarzhans W, Jaramillo C. An exceptional marine fossil fish assemblage reveals a highly productive deep-water environment in the Central American Seaway during the late Miocene. Geol Soc Am Abs. 2012; 44: 164.

[47] Fierstine HL. A new marlin, *Makaira panamensis*, from the Late Miocene of Panama. Copeia. 1978; 1978: 1–11.

[48] Schwarzhans W, Aguilera O. Otoliths of the Myctophidae from the Neogene of tropical America. Palaeo Ichthyologica. 2013; 13: 83–150.

[49] Fitch JE, Brownell RL Jr. Fish otoliths in cetacean stomachs and their importance in interpreting feeding habits. J Fish Board Can. 1968; 25: 2561–2574.

[50] Staudinger MD, McAlarney RJ, McLellan WA, Pabst DA. Foraging ecology and niche overlap in pygmy (*Kogia breviceps*) and dwarf (*Kogia sima*) sperm whales from waters of the U.S. mid-Atlantic coast. Mar Mamm Sci. 2014; 30: 626–655.

[51] Lindberg DR, Pyenson ND. Things that go bump in the night: evolutionary interactions between cephalopods and cetaceans in the Tertiary. Lethaia. 2007; 40: 335–343.

[52] Werth AJ. Mandibular and dental variation and the evolution of suction feeding in Odontoceti. J Mamm. 2006; 87: 579–588.

[53] Lucas SG, McLeod SA, Barnes LG, Alvarado GE, García R, Espinoza E. A baleen whale from the Pliocene of Nicaragua. Rev Geol Am Cen. 2009; 41: 14–24.

[54] Laurito CA, Valerio AL, Hernández AC, Ovares E. Primer registro de un cetáceo fossil (Mammalia, Cetacea, Odontoceti, Squalodontidae) en la Formación Río Banana, Mioceno medio de Costa Rica, América Central. Rev Geol Am Cen. 2011; 44: 153–156.

[55] Valerio AL, Laurito CA. Cetáceos fósiles (Mammalia, Odontoceti, Eurhinodelphinoidea, Inioidea, Physeterioidea) de la Formación Curré, Mioceno superior (Hemphilliano temprano tardío) de Costa Rica. Rev Geol Am Cen. 2012; 46: 151–160.

[56] Uhen MD, Coates AG, Jaramillo CA, Montes C, Pimiento C, Rincon A, et al Marine mammals from the Miocene of Panama. J S Am Earth Sci. 2010; 30: 167–175.

[57] Velez-Juarbe J, Wood A, Ridgwell N, Bloch J, MacFadden B. Partial skeleton of a toothed whale (Odontoceti, Cetacea) from the mid to late Miocene Gatun Formation, Panama. J Vertebr Paleontol Progr Abstr. 2013: 231.

[58] Vigil DI, Laurito CA. Nuevos restos de un Odontoceti fósil (Mammalia: Cetacea, Physeteroidea) para el Mioceno tardío de Panamá, América Central. Rev Geol Am Cen. 2014; 50: 213–217.

[59] Morgan GS. Miocene and Pliocene marine mammal faunas from the Bone Valley Formation of Central Florida. Proc San Diego Soc Nat Hist. 1994; 29: 239–268.

[60] Barnes LG. Fossil odontocetes (Mammalia: Cetacea) from the Almejas Formation, Isla Cedros, Mexico. PaleoBios. 1984; 42: 1–46.

[61] Barnes LG. Miocene and Pliocene Albireonidae (Cetacea, Odontoceti), rare and unusual fossil dolphins from the eastern North Pacific Ocean. Nat Hist Mus Los Angeles County Sci Ser. 2008; 41: 99–152.

[62] Pyenson ND, Gutstein CS, Parham JF, Le Roux JP, Carreño Chavarría C, Metallo A, et al Repeated mass strandings of Miocene marine mammals from the Atacama Region of Chile point to sudden death at sea. Proc R Soc B. 2014; 281: 20133316.s 10.1098/rspb.2013.3316 24573855 PMC3953850

