# Correction: Evolutionary Patterns among Living and Fossil Kogiid Sperm Whales: Evidence from the Neogene of Central America

**DOI:** 10.1371/journal.pone.0129186

**Published:** 2015-05-20

**Authors:** Jorge Velez-Juarbe, Aaron R. Wood, Carlos De Gracia, Austin J. W. Hendy

The affiliation for the second author is incorrect. Aaron R. Wood is affiliated with #3 and with #6: Florida Museum of Natural History, University of Florida, Gainesville, Florida, United States of America.

The Funding section is incorrect. The correct funding information is: Funding for this project was provided by National Science Foundation Partnerships for International Research and Education grant #0966884; National Science Foundation Earth Sciences Postdoctoral Fellowship grant #1249920 to JVJ; and Secretaria Nacional de Ciencia, Tecnología en Innovación grant #APY-NI10-016A to CDG. The funders had no role in study design, data collection and analysis, decision to publish, or preparation of the manuscript.
