# Supplementary material for: Evolutionary Patterns among Living and Fossil Kogiid Sperm Whales: Evidence from the Neogene of Central America
Source: PLoS One. 2015 Apr 29;10(4):e0123909. doi: 10.1371/journal.pone.0123909 (PMC4414568; doi:10.1371/journal.pone.0123909)
Supplement: S1 Dataset — (DOC) [file pone.0123909.s001.doc]

Electronic Supplementary Material (ESM) 1 for:

**Evolutionary patterns among living and fossil kogiid sperm whales: evidence from the Neogene of Central America**

**Jorge Velez-Juarbe^1,2*^, Aaron R. Wood^3^, Carlos De Gracia^4^, and Austin J. W. Hendy^5^**

^1^*Department of Mammalogy, Natural History Museum of Los Angeles County, Los Angeles, California, United States of America*

^2^*John D. Cooper Archaeological and Paleontological Center, Department of Geological Sciences, California State University, Fullerton, California, United States of America*

^3^*Department of Geological and Atmospheric Sciences, Iowa State University, Ames, Iowa, United States of America*

^4^*Smithsonian Tropical Research Institute, Balboa-Ancon, Panama*

^5^*Department of Invertebrate Paleontology, Natural History Museum of Los Angeles County, Los Angeles, California, United States of America*

**Author for correspondence (jvelezjuar@nhm.org) (JVJ).*

**Supplementary material 1:** List of characters and states, and matrix used in the phylogenetic analysis.

Table of Content:

1. Character-State List p. S3

2. Matrix p. S7

3. References p. S10

**1. List of Characters and States**

The characters used in the phylogenetic analysis (based on Lambert et al. [1]). The list consists of 41 multistate morphological characters; they are treated as unordered.

1. *Rostrum length*: (0) rostrum elongated, ratio between rostrum length and skull width > 1.2; (1) ratio ≤ 1.2 and ≥ 0.95; (2) short rostrum, ratio > 0.95.

2. *Maxillae, premaxillae and vomer, all reaching the tip of the rostrum which is not formed only by the premaxillae*: (0) absent; (1) present.

3. *Supracranial basin of the skull*: (0) absent; (1) present; (2) extended onto the whole dorsal surface of the rostrum.

4. *Dorsal exposure of the maxilla on the rostrum*: (0) exposure limited to less than half the rostrum length; (1) maxilla exposed on more than half the length of the rostrum, narrower than the premaxilla at some levels; (2) wider than the premaxilla all along.

5. *Constriction of premaxilla anterior to antorbital notch followed by anterior expansion*: (0) absent, suture maxilla-premaxilla on the rostrum roughly anteriorly directed; (1) present, suture maxilla-premaxilla distinctly anterolaterally directed.

6. *Upper tooth row*: (0) present; (1) absent.

7. *Premaxillary teeth*: (0) present; (1) absent.

8. *Maximum width of skull (postorbital or bizygomatic width)*: (0) < 40 cm; (1) ≥ 40 and < 60 cm; (2) ≥ 60 and < 100 cm; (3) ≥ 100 cm.

9. *Antorbital notch*: (0) absent; (1) present; (2) transformed into a very narrow slit.

10. *Antorbital notch*: (0) outside the supracranial basin; (1) inside the supracranial basin.

11. *Number and size of dorsal infraorbital foramina, in the area of the right antorbital notch and posteriorly*: (0) small to moderate size foramina, at least three-four; (1) three large foramina; (2) two large foramina; (3) one large foramen (maxillary incisure).

12. *Right premaxilla*: (0) posteriorly extended as the left premaxilla; (1) more posteriorly extended than the left premaxilla.

13. *Right premaxilla*: (0) not widened posteriorly; (1) posterior extremity of the right premaxilla laterally widened, occupying at least one third of the width of the supracranial basin.

14. *Presence of a sagittal crest*: (0) absent; (1) present as a shelf covered by the pointed right premaxilla.

15. *Left premaxillary foramen very small or absent*: (0) absent; (1) present.

16. *Increase in size of the right premaxillary foramen*: (0) absent, ratio between width of foramen and width of premaxilla at that level ≤ 0.20; (1) present, ratio > 0.20.

17. *Anteroposterior level of right premaxillary foramen*: (0) distinctly anterior to antorbital notch; (1) slightly anterior to antorbital notch; (2) same level or posterior to antorbital notch.

18. *Asymmetry of the bony nares*: (0) absent or reduced; (1) strong, left bony naris significantly larger than right naris.

19. *Lack of nasals*: (0) both nasals present; (1) one nasal absent; (2) both nasals absent.

20. *Widening of the supracranial basin on the right side*: (0) absent; (1) present, basin overhangs the right orbit.

21. *Right maxilla reaching the sagittal plane of the skull on the posterior wall of the supracranial basin*: (0) absent; (1) present.

22. *Fusion of lacrimal and jugal*: (0) absent; (1) present.

23. *Projection of the lacrimal-jugal between frontal and maxilla*: (0) short or absent; (1) long.

24. *Preorbital process considerably lower than the elevated dorsolateral margin of the rostrum base*: (0) absent; (1) present.

25. *Frontal-maxilla suture, with skull in lateral view*: (0) forming an angle < 15º from the axis of the rostrum; (1) 15-35º; (2) > 35º.

26. *Temporal fossa*: (0) anteroposteriorly longer than distance between preorbital process of the maxilla and anterior wall of temporal fossa; (1) approximately same length; (2) distinctly shorter.

27. *Zygomatic process of squamosal in lateral view*: (0) ‘L’-shaped with dorsal margin ventrally bending in its posterior portion; (1) triangular, with dorsal margin dorsally bending in its posterior portion.

28. *Postglenoid process of the squamosal*: (0) significantly ventrally longer than post-tympanic process; (1) roughly same ventral extent as post-tympanic process.

29. *In lateral view of the skull, wide notch posterior to the postglenoid process of the squamosal for the enlarged posterior process of the tympanic*: (0) absent; (1) present.

30. *Occipital shield*: (0) convex and forming an angle of about 40º from the axis of the rostrum; (1) as state 0 with an angle of about 60º; (2) flat or concave forming an angle of about 90º.

31. *Long axis of the skull*: (0) roughly parallel to the long axis of the body (perpendicular to the surface of the occipital condyles); (1) projected ventrally.

32. *Falciform process of the squamosal*: (0) contacting the corresponding pterygoid; (1) forming a thin plate not contacting the pterygoid; (2) reduced to a simple peg or absent.

33. *Anterior bullar facet of the periotic*: (0) very anteroposterioly elongated; (1) reduced; (2) absent or very small.

34. *Posterior extension of the posterior process of the periotic parallel to the general plane of the bone and not ventrally oriented*: (0) absent; (1) present.

35. *Accessory ossicle of the tympanic bulla*: (0) absent; (1) present; (2) present and partially fused with the anterior process.

36. *Involucrum of the tympanic bulla with an evident central concavity, visible in ventral and medial views, due to the marked pachyostosis of its anterior and posterior portions*: (0) absent; (1) present.

37. *Size of teeth (greatest transverse diameter of root expressed as percentage of the maximum width of skull)*: (0) < 5%; (1) > 5%.

38. *Loss of dental enamel*: (0) absent; (1) present.

39. *Number of mandibular teeth*: (0) 11; (1) 12-14; (2) > 14.

40. *Labiolingual compression of the posterior lower teeth (portion out of the alveolus)*: (0) strong; (1) weak or absent.

41. *Ventral position of the mandibular condyle*: (0) absent, well developed angular process; (1) present, angular process low or absent.

**2. Character-Taxon Matrix**

The matrix used here is based n that published by Lambert et al. [1] for Physeteroidea, with the additions of *Hesperokogia tyrioni*, *Kogia prisca*, *K. sima*, and *K. breviceps* (previous analyses used *Kogia* spp. as a single operational unit). The list includes 2 outgoup and 18 ingroup taxa; all characters were left unordered and of equal weight.

*Zygorhiza kochii*

0 0 0 0 0 0 0 0 0 - - 0 0 0 - - - 0 0 - - 0 0 0 ? 0 0 0 0 2 0 0 0 0 0 0 0 0 0 0 0

*Agorophius pygmaeus*

? ? 0 1 0 0 ? 0 1 - 0 0 0 0 0 0 0 0 0 - 0 ? 0 0 0 0 0 0 0 0 0 ? ? ? ? ? ? ? ? ? ?

*Eudelphis mortezelensis*

1 0 1 ? 0 0 ? 1 1 0 0 1 ? ? 0 1 ? 1 ? 0 ? ? ? ? ? ? 1 0 0 ? 0 1 ? ? ? ? 0 0 ? ? ?

*Zygophyseter varolai*

1 0 1 1 0 0 1 2 1 0 2 1 1 0 1 1 2 1 1 1 ? 1 0 0 1 0 1 1 0 ? 0 1 2 ? 2 1 1 0 1 0 1

*Brygmophyseter shigensis*

? ? 1 ? ? 0 ? 2 1 0 2 1 1 0 1 ? ? 1 ? ? ? ? ? 0 1 0 1 1 0 1 ? ? ? ? ? ? 1 0 1 ? 1

*Livyatan melvillei*

2 1 2 1 1 0 1 3 2 0 2 ? 1 0 0 ? ? ? ? 0 0 1 ? 0 2 0 1 ? 0 ? ? 2 ? ? ? ? 1 0 0 1 1

*Placoziphius duboisi*

? 0 1 2 0 ? ? 1 1 0 ? 1 1 0 1 1 1 ? 1 0 ? ? 0 0 1 ? 1 1 0 ? ? 2 ? ? ? ? ? 0 ? ? ?

*Orycterocetus crocodilinus*

1 0 1 1 0 0 0 1 1 0 1 1 1 0 1 0 1 1 1 0 1 ? 0 0 1 1 1 1 0 2 0 2 2 0 2 1 0 0 2 ? ?

*Physeterula dubusi*

0 ? 1 0 ? 0 ? 2 1 0 2 1 1 0 1 ? ? 1 ? 0 0 ? ? 0 1 ? 1 1 0 2 ? 2 ? ? ? ? 0 ? 2 1 ?

*Aulophyseter morricei*

1 0 1 0 0 1 - 2 1 0 2 1 1 0 1 0 2 1 1 0 0 1 0 1 2 2 1 1 0 2 ? ? 2 0 2 ? 0 ? ? ? ?

*Acrophyseter deinodon*

2 0 1 1 0 0 0 1 ? 0 3 ? 1 0 0 1 1 1 ? 1 ? ? ? 0 2 0 1 1 0 1 0 ? ? ? ? ? 1 0 1 0 1

*Physeter macrocephalus*

0&1 0 2 2 1 1 - 3 1 0 3 1 1 0 1 0 2 1 1 0 0 1 0 1 2 2 1 1 0 2 1 2 2 0 2 1 0 1 2 1 0&1

*Thalassocetus antwerpiensis*

? ? ? ? ? ? ? 0 1 0 1 1 0 1 ? ? ? ? 2 0 1 ? ? 0 1 ? 1 1 0 1 ? ? ? ? ? ? ? ? ? ? ?

*Scaphokogia cochlearis*

? 1 1 1 1 1 - 0 2 0 0 1 0 1 1 0 2 1 2 0 ? 1 1 1 1 ? ? ? ? ? 1 ? 2 1 2 ? ? 1 ? ? ?

*Praekogia cedrosensis*

? ? 1 ? ? ? ? 0 2 1 0 1 0 1 ? ? ? 1 2 0 1 ? 1 ? 1 1 1 0 1 1 1 2 ? ? ? ? ? ? ? ? ?

*Kogia sima*

2 1 2 2 0 1 - 0 2 1 0 1 0 1 1 0 1&2 1 2 0 1 1 1 0 2 2 1 0 1 1&2 1 2 2 1 2 1 0 1 0&1&2 1 0&1

*Kogia breviceps*

2 1 2 2 0 1 - 0 2 1 0 1 0 1 1 0 1&2 1 2 0 1 1 1 0 2 2 1 0 1 1&2 1 2 2 1 2 1 0 1 0&1&2 1 0&1

*Kogia pusilla*

2 1 2 2 0 1 - 0 2 1 ? 1 0 1 ? ? ? 1 2 0 1 1 1 0 1 ? ? ? ? ? ? ? ? ? ? ? ? ? ? ? ?

*Aprixokogia kelloggi*

? ? 2 ? ? 0 ? 0 1 0 0 1 1 1 1 0 2 1 2 0 1 1 1 1 1 0 1 1 0 2 1 2 ? ? ? ? ? 1 ? ? ?

*Nanokogia isthmia* gen. et sp. nov.

2 1 1 1 0 1 - 0 2 1 0 1 0 1 0 ? ? 1 2 0 1 1 0 0 1 0 1 0 1 1 1 2 ? ? ? ? 0 ? 1 1 ?

**3. References**

1. Lambert O, Bianucci G, Post K, de Muizon C, Salas-Gismondi R, Urbina M, et al. The giant bite of a new raptorial sperm whale from the Miocene epoch of Peru. Nature. 2010; 466: 105–108.
