# Supplementary figures and images for: Evolutionary Patterns among Living and Fossil Kogiid Sperm Whales: Evidence from the Neogene of Central America
Source: PLoS One. 2015 Apr 29;10(4):e0123909. doi: 10.1371/journal.pone.0123909 (PMC4414568; doi:10.1371/journal.pone.0123909)

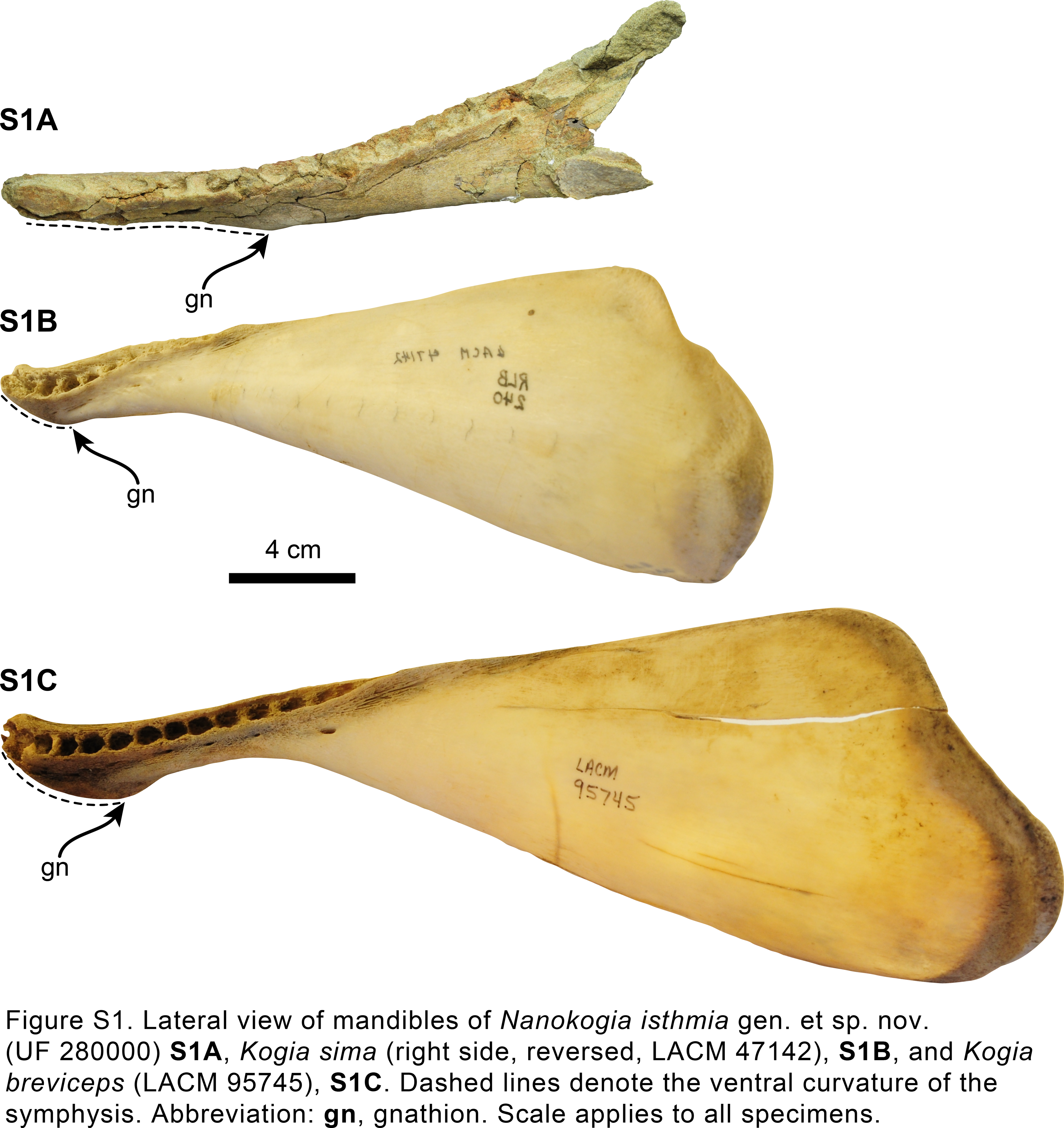

Supplement: S1 Fig — Lateral views of mandibles of Nanokogia isthmia gen. et sp. nov. (UF 280000) S1A, Kogia sima (right side, reversed, LACM 47142), S1B, and Kogia breviceps (LACM 95745), S1C. Dashed lines denote the ventral curvature of the symphysis. Abbreviations: gn, gnathion. Scale applies to all specimens. (TIF) [file pone.0123909.s002.tif]

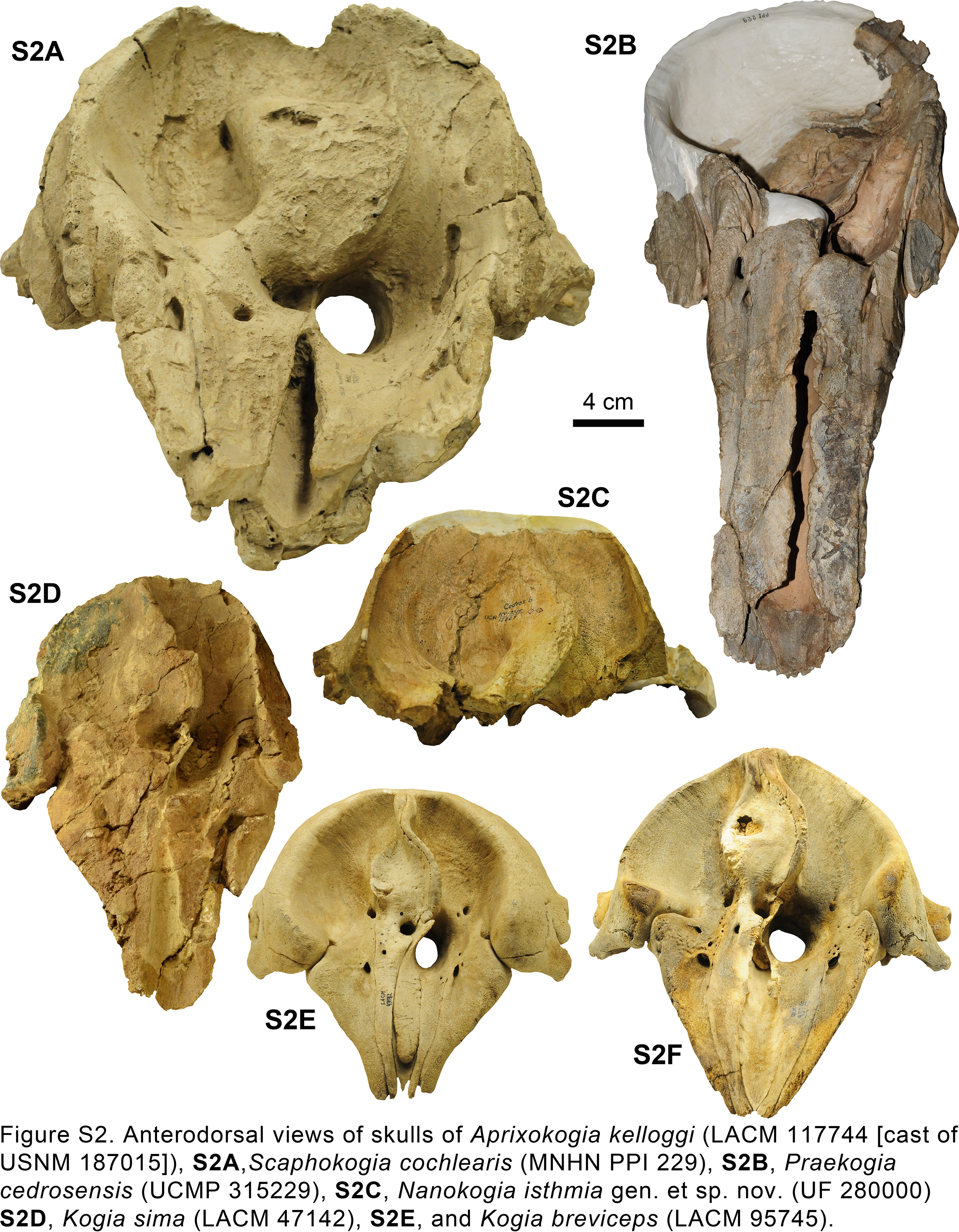

Supplement: S2 Fig — Anterodorsal views of skulls of Aprixokogia kelloggi (LACM 117744 [cast of USNM 187015]), S2A, Scaphokogia cochlearis (MNHN PPI 229), S2B, Praekogia cedrosensis (UCMP 315229), S2C, Nanokogia isthmia gen. et sp. nov. (UF 280000), S2D, Kogia sima (LACM 47142), S2E, and Kogia breviceps (LACM 95745). (TIF) [file pone.0123909.s003.tif]

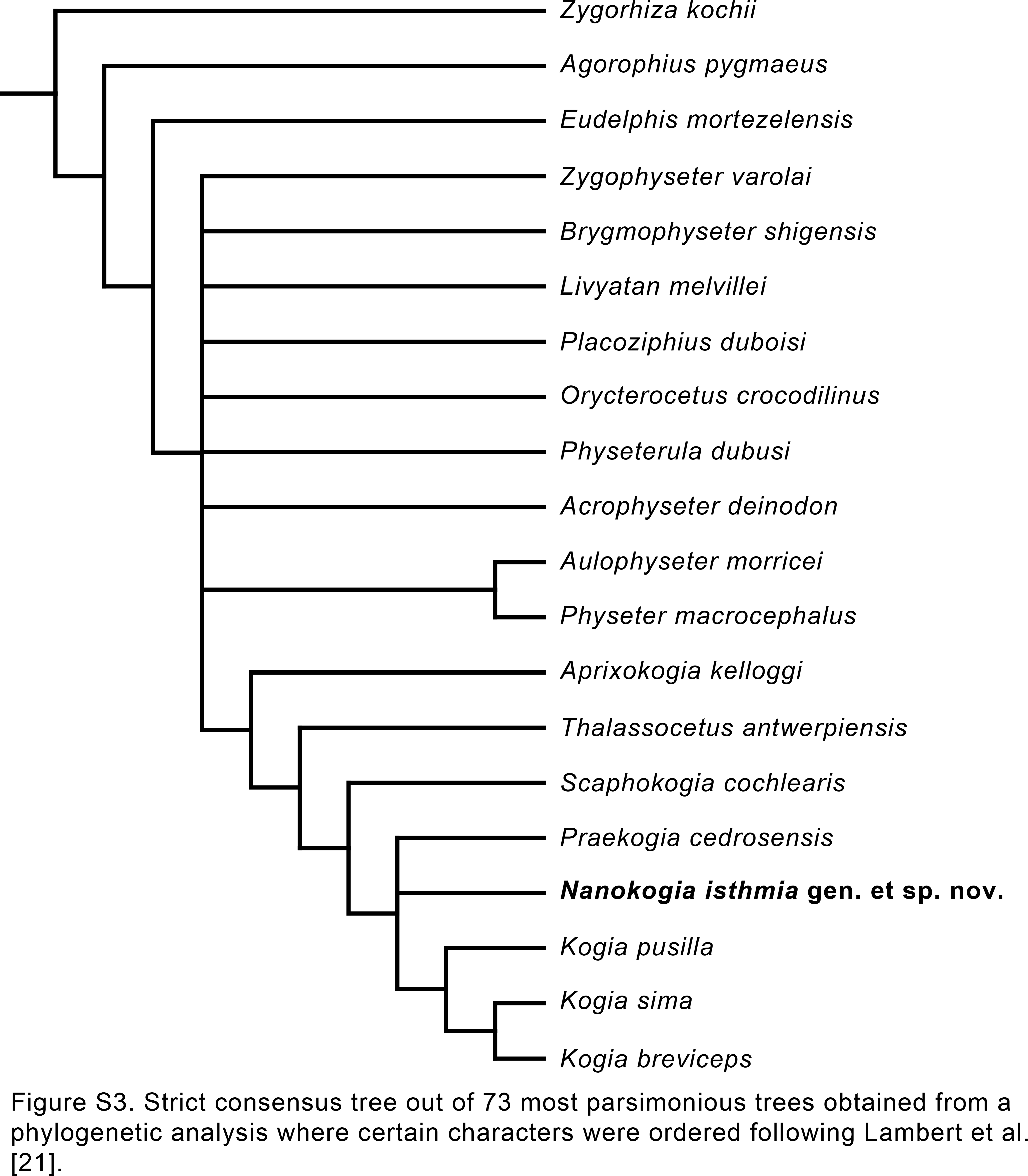

Supplement: S3 Fig — Strict consensus tree out of 73 most parsimonious trees obtained from a phylogenetic analysis where certain characters were ordered following Lambert et al. [21]. (TIF) [file pone.0123909.s004.tif]
